# An Open and Novel Low-Cost Terrestrial Laser Scanner Prototype for Forest Monitoring

**DOI:** 10.3390/s26010063

**Published:** 2025-12-21

**Authors:** Jozef Výbošťok, Juliána Chudá, Daniel Tomčík, Dominik Gretsch, Julián Tomaštík, Michał Pełka, Janusz Bedkowski, Michal Skladan, Martin Mokroš

**Affiliations:** 1 Department of Forest Harvesting, Logistics and Ameliorations, Technical University in Zvolen, T. G. Masaryka 24, 96001 Zvolen, Slovakia; xchudaj@is.tuzvo.sk (J.C.); xtomcikd@is.tuzvo.sk (D.T.); xgretschd@is.tuzvo.sk (D.G.); xskladan@is.tuzvo.sk (M.S.); 2 Department of Forest Resource Planning and Informatics, Technical University in Zvolen, T. G. Masaryka 24, 96001 Zvolen, Slovakia; tomastik@tuzvo.sk; 3 Institute of Fundamental Technological Research, Polish Academy of Science, ul. Pawi ńskiego 5B, 02-106 Warsaw, Poland; michalpelka@gmail.com (M.P.); jbedkows@ippt.pan.pl (J.B.); 4 Department of Geography, University College London, Gower Street, London WC1E 6BT, UK; m.mokros@ucl.ac.uk

**Keywords:** functional prototype, low cost, TLS, HMLS, iPhone, forest

## Abstract

**Highlights:**

**What are the main findings?**

- The low-cost TLS prototype was constructed for less than EUR 2050.
- All hardware components are publicly available, enabling easy construction.

**What are the implications of the main findings?**

- Full build documentation will be shared on Zenodo.
- The LCA-TLS prototype, based on the Livox Avia sensor, accurately estimates key dendrometric parameters such as DBH and tree height

**Abstract:**

Accurate and efficient forest inventory methods are crucial for monitoring forest ecosystems, assessing carbon stocks, and supporting sustainable forest management. Traditional field-based techniques, which rely on manual measurements such as diameter at breast height (DBH) and tree height (TH), remain labour-intensive and time-consuming. In this study, we introduce and validate a fully open-source, low-cost terrestrial laser scanning system (LCA-TLS) built from commercially available components and based on the Livox Avia sensor. With a total cost of €2050, the system responds to recent technological developments that have significantly reduced hardware expenses while retaining high data quality. This trend has created new opportunities for broadening access to high-resolution 3D data in ecological research. The performance of the LCA-TLS was assessed under controlled and field conditions and benchmarked against three reference devices: the RIEGL VZ-1000 terrestrial laser scanner, the Stonex X120GO handheld mobile laser scanner, and the iPhone 15 Pro Max structured-light device. The LCA-TLS achieved high accuracy for estimating DBH (RMSE: 1.50 cm) and TH (RMSE: 0.99 m), outperforming the iPhone and yielding results statistically comparable to the Stonex X120GO (DBH RMSE: 1.32 cm; *p* > 0.05), despite the latter being roughly ten times more expensive. While the RIEGL system produced the most accurate measurements, its cost exceeded that of the LCA-TLS by a factor of about 30. The hardware design, control software, and processing workflow of the LCA-TLS are fully open-source, allowing users worldwide to build, modify, and apply the system with minimal resources. The proposed solution thus represents a practical, cost-effective, and accessible alternative for 3D forest inventory and LiDAR-based ecosystem monitoring.

## 1. Introduction

Forest ecosystems play a key role in addressing climate change, conserving biodiversity, and providing a wide range of ecosystem services [1,2,3]. Detailed monitoring based on accurate data is essential to ensure effective and sustainable management of these ecosystems. Forest field inventory plays a crucial role in assessing forest resources by collecting detailed measurements of tree characteristics within sample plots. To monitor and manage ecosystems, forest inventory is a fundamental process for collecting necessary data on tree structure and composition, leaning on the main tree characteristics: the number of the trees, diameter at breast height (DBH), and tree height (TH) [4,5].

Most essential characteristics of forest stands, such as density or structural composition, are primarily based on the number of trees within a given area. However, tree count alone offers only a partial view of stand condition. Only when combined with DBH and TH can a comprehensive understanding of forest volume, structure, and production potential be achieved. These fundamental parameters are therefore inseparable and form the foundation of any reliable forest ecosystem assessment [6,7]. However, collecting such data in the field, although essential for accurately assessing forest condition, is often challenging due to the continued reliance on conventional methods that have seen little innovation in recent decades. This highlights the need for more efficient and innovative approaches to forest inventory [8,9,10]. Conventional methods typically require measuring the DBH for every tree and estimating the height of selected trees. Tree height is most commonly measured using altimeters, while DBH is typically assessed with callipers or diameter tapes. Although these traditional methods are widely used in forestry, they are time-consuming, labour-intensive, and economically demanding due to the high costs associated with the required human labour, despite the methods themselves being relatively inexpensive. Therefore, the traditional approaches are often limited in efficiency for large-scale applications [11,12].

Moreover, these methods are insufficient to meet the growing demand for detailed, large-scale forest information. This highlights the necessity of developing new approaches for quantifying dendrometric parameters at both the individual tree and ecosystem levels.

The use of LiDAR-based technologies that generate 3D point clouds is a promising option. In general, LiDAR systems can be categorized as either airborne or ground-based. This study focuses exclusively on ground-based LiDAR systems and does not address airborne platforms. Among ground-based systems, three main types exist: terrestrial laser scanners (TLSs), mobile laser scanners (MLSs), and smart devices equipped with time-of-flight sensors (SLDs). TLS was first to be employed among these technologies and has therefore been the most extensively tested and validated in various research settings starting in the early 2000s [13].

MLS has been increasingly applied in forest environments since the early 2010s, initially relying on backpack-type or vehicle-mounted systems that integrated GNSS and IMU components. However, dense forest canopies often cause signal degradation and multipath errors, leading to misaligned point clouds and reduced positional accuracy [14,15]. For example, Tang et al. (2015) [15] reported a 38% improvement in positional accuracy using SLAM over conventional GNSS/IMU-based methods. Similarly, authors achieved a 70–86% improvement in stem mapping accuracy by implementing heading/velocity-aided SLAM [16]. Due to these challenges, the development of MLS systems shifted towards more compact, handheld solutions equipped with Simultaneous Localization and Mapping (SLAM) algorithms, which allow for real-time mapping without dependence on external GNSS signals. These devices, such as the Emesent Hovermap or Kaarta Stencil 2, have demonstrated root mean square errors (RMSEs) for DBH estimation ranging from 1.21 cm to 5.57 cm and tree height RMSE as low as 0.42 m [17,18,19]. Such systems have proven particularly suitable for forest inventory in environments where satellite signal availability is limited.

The most recent advancement in scanning technology is the integration of time-of-flight sensors into smart devices. While several manufacturers have explored similar concepts of mobile spatial sensing, for example, the use of Google Tango devices combining RGB-D, inertial sensors and SLAM for forest inventory measurements [20], Apple has been systematically implementing such sensors in its devices starting in 2020 with the iPad Pro, iPhone 12 Pro, and iPhone 12 Pro Max. And it continues with all-new Pro and Pro Max models. In forestry applications, these devices were first used as early as 2021 [21], demonstrating their potential as a novel, low-cost solution.

Every device comes with its own set of strengths and limitations, which affect how and where it can be effectively used. While TLS enables precise and high-resolution spatial data [22], it is also the most costly and time-intensive method for data collection [10]. Compared to TLS, handheld mobile laser scanner (HMLS) offers advantages in cost and speed, but it does not reach the same level of accuracy [23]. Among the tested devices, the iPhone is the most affordable of all the devices. However, its main limitations are the short sensor range, approximately 5 m [24], and the lowest DBH estimation accuracy among the devices evaluated, with an RMSE of 2.27 cm [21].

Two primary types of terrestrial LiDAR technologies are used: time-of-flight (ToF) and phase-shift scanners. While both are effective for capturing forest structure, phase-shift systems often used in TLS have demonstrated superior accuracy, with relative RMSE values of 3.7–6.4% for tree parameters. In comparison, ToF systems, including consumer-grade devices like the iPad Pro, show higher error rates of 8.6–12.9% [21].

Beyond the physical measurement principle, the temporal dimension of scanning is also critical. In repetitive scanning, the same forest area is surveyed multiple times over months or years, enabling detailed change detection for ecological monitoring, such as tracking biomass development or structural changes [25,26]. On the other hand, non-repetitive scanning is used for one-time data capture, as is common in forest inventories and baseline mapping campaigns [27,28]. A key enabler for both one-time and repeated scanning approaches is the rise of non-spinning LiDAR systems. These devices replace mechanical rotation with solid-state or MEMS-based (microelectromechanical systems) beam steering, resulting in smaller, lighter, and more robust sensors. Such advancements have been made possible by progress in photonics, miniaturization, and the integration of IMU, GNSS, and SLAM components [25]. Consequently, these technological improvements have also contributed to a substantial reduction in LiDAR system costs. The use of consumer-grade electronics (e.g., iPad Pro, Leica BLK360), combined with mobile and handheld designs and the availability of sub-$20,000 devices, has made high-resolution forest data collection feasible even for smaller research teams and operational forestry [27,28,29]. Lower software costs and open-source processing tools further enhance accessibility, marking a shift toward more scalable and affordable terrestrial LiDAR workflows.

Anecdotal trends based on publication search queries such as “TLS + Forest” versus “MLS OR HMLS + Forest” indicate that while traditional TLS applications in forestry may have peaked, interest in mobile and handheld systems continues to rise. This shift is likely driven by the high operational costs and logistical complexity of conventional TLS setups. However, the recent emergence of low-cost TLS systems, especially those using non-repetitive, compact designs, offers a potential second wave of adoption. By significantly lowering the cost and technical barriers, these systems can make TLS more accessible for practical forest inventory tasks, particularly in contexts where repeatability, spatial accuracy, and 3D detail remain essential, but budgets and manpower are limited.

For that reason, the goal of the research presented in this paper was to develop a low-cost terrestrial laser scanner (LCA-TLS) with an overall cost below 3000 euros and its suitability for forest inventory applications. The core research question underlying this study is whether a low-cost, fully open-source LCA-TLS can achieve accuracy levels that are sufficient for key forest inventory parameters such as DBH and tree height. A key advantage of the proposed prototype is that all its components are publicly available, enabling its construction with standard equipment. The most significant outcomes of this study were the creation of a fully functional terrestrial laser scanner, including the assessment of the prototype’s performance and the demonstration of the feasibility of using an LCA-TLS based on the Livox Avia sensor to determine fundamental dendrometric parameters such as DBH and tree height. We have also conducted a performance evaluation and comparison of the LCA-TLS system with conventional TLS, HMLS, and SLD sensors in terms of accuracy, time efficiency, and cost-effectiveness. The devices were initially tested in laboratory conditions to verify the functionality of the systems and then tested in a forest/plantation environment.

## 2. The Development of a Prototype of a Low-Cost Terrestrial Laser Scanner (LCA-TLS)

Two important parts of prototype development are hardware and software. Here, we will introduce both and give the reader the pipeline on how to develop such a terrestrial laser scanner that will operate but also have functional software to control the scanner and store point clouds, which will be ready for further processing. The documentation and guidelines, together with the .stl files for 3D printing, are available at https://zenodo.org/records/16531534 (accessed on 28 July 2025).

### 2.1. Hardware

**Figure 1:**
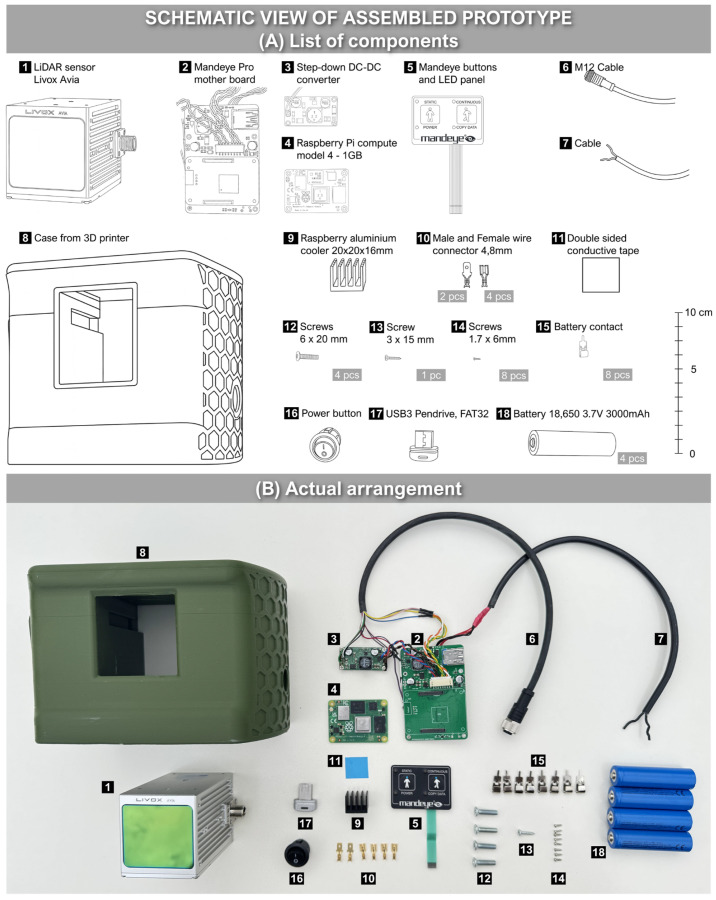
Schematic view of LCA-TLS prototype.

The proposed low-cost TLS consists of commercially available components that can be purchased. The LCA-TLS consists of the following (Figure 1A):

- LiDAR sensor Livox Avia.
- M12 cable.
- Power connector.
- Power button.
- Raspberry Pi CM4.
- Mandeye Pro motherboard.
- Mandeye buttons and LED panel.
- Aluminum cooler.
- Cables.
- Battery.
- Step-down DC-DC converter.

The LCA-TLS system is powered by four serially connected 18,650 batteries, each rated at 3.7 V and 3000 mAh. Although Livox Avia accepts a maximum of 15 V, a fully charged 4S Li-Ion battery pack can provide almost 17.0 V. Therefore, a step-down DC-DC converter is required.

All components were placed in a 3D enclosure created by the authors and then printed on a 3D printer Prusa XL using a thermoplastic polyester PTEG (Polyethylene Terephthalate Glycol) (Figure 1B). A total of 860 g of material was utilized for the prototype.

*Livox Avia sensor*

LIVOX AVIA is a lidar sensor based on incommensurable scanning (non-repetitive scanning pattern) that allows for adoption in plenty of applications such as autonomous robots, mobile mapping, security, etc. [1], and is capable of recording up to three returns per laser pulse. The incommensurable scanning method of this lidar can potentially provide higher resolution than conventional lidars, which is beneficial in mobile mapping applications. The disadvantage of this model is a narrow field of view, both in non-repetitive scanning pattern (70.4°: horizontal × 77.2°: vertical) and repetitive line scanning (70.4°: Horizontal × 4.5°: vertical) (Table 1). The sensor is specified by the manufacturer to operate in the temperature range −20 °C to +65 °C and features an IP67 rating (sensor body), which suggests dust- and water-resistance under moderate exposure. However, this rating applies only to the sensor itself.

**Figure 2:**
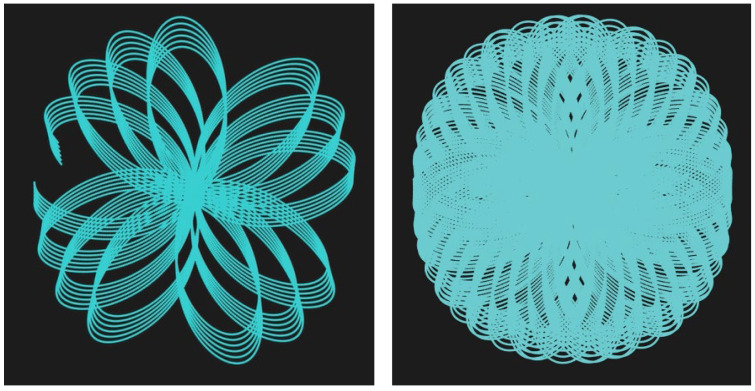
Incommensurable scanning (non-repetitive scanning pattern) of LIVOX AVIA. (**Left**): small number of scanning measurements. (**Right**): aggregated large number of scanning measurements (source https://www.livoxtech.com/avia/specs (accesses on 8 October 2025)).

Figure 2 shows the scanning pattern in two different situations: as a small number of scanning measurements (small time of data acquisition) and as an aggregated large number of scanning measurements. It can be observed that the coverage of the measured 3D space is growing over time, even though we do not move the sensor. This feature is the main advantage of our TLS prototype since we control scanning resolution by the time period of the acquisition. The scanner provides 240,000 points per second based on the first/strongest return, thus potentially reaching up to 1,000,000 points/s.

*Assembly of Prototype*

**Figure 3:**
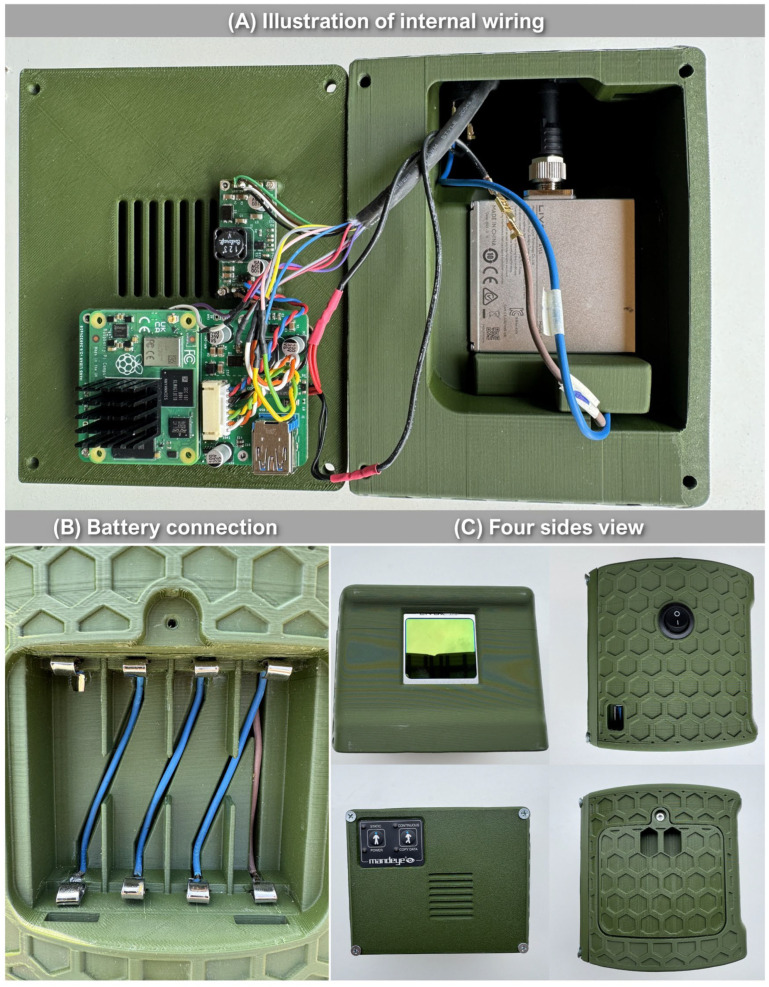
Device architecture and layout.

The entire prototype relies on readily accessible parts (Figure 3), making it possible for anyone to build it under home conditions. Besides the components, it is necessary to be equipped with a soldering iron and a 3D printer. As mentioned, all documentation required to construct the prototype is available on Zenodo at the following link: https://doi.org/10.5281/zenodo.16531534 (accessed on 28 July 2025). The entire system (except for Livox Avia) is based on open hardware developed by the authors of this article. The weight of the prototype is 1.5 kg.

### 2.2. Software

The software runs at Single Board Computer (SBC, Raspberry Pi Ltd., Cambridge, UK) inside the LCA-TLS prototype as a service. It operates under a Linux distribution (Raspbian Bullseye, based on Debian 11; released on 14 August 2021) officially supported for the SBC platform. The software developed by the authors is written in C++, distributed under a permissive MIT license, and is fully open-source and publicly available at https://github.com/JanuszBedkowski/mandeye_controller/tree/livox_sdk_1 (accessed on 5 February 2025).

It utilizes multiple libraries:

- SDK from Lidar’s manufacturer (Livox Technology Company Limited Co., Ltd., Shenzhen, China) is used to configure and obtain data from Lidar range measurements and IMU.
- GPIOd library from Linux kernel is used to read and write GPIO (General purpose input-outputs) of the SBC.
- Pistache for web interface and debugging.
- LASzip—a library for compressing and saving point cloud in LAZ format.

The software running on the LCA-TLS is a state machine with multiple states that the user can change using buttons. The state of the device is shown in the LED panel. There are two states for scanning (continuous and stop-scan) and multiple transition steps (e.g., for finalizing data). Some states result in errors (e.g., problems with USB or LiDAR).

The service is a multi-threaded C++ executable, where the main thread is responsible for running the state machine and separate threads for data acquisition (LiDAR, IMU, and optional GNSS). When the state is changed to scanning, the data acquisition threads start collecting data into a growing buffer. After 10 s the main thread obtains buffers from all threads using move semantic.

Next, LASzip routines are called to compress point cloud data and save them to the USB pendrive. Other data are not saved without compression. There is a heuristic algorithm that prevents the explosion of memory usage if the compression and saving time is too long. This time-based heuristic triggers on-the-fly decimation in such a situation. The architecture is an example of a producer–consumer model.

## 3. Materials and Methods

We have established two main experiments to assess the prototype’s performance. Firstly, we have created an environment within a laboratory where we have placed tubes of various sizes. The main reason for using lab conditions was to focus primarily on the noise of the scanners, where we will not have other influencing factors, for example, movements of trees and branches caused by wind. Then, we performed an experiment within the tree plantation of fast-growing trees. Both environments have also been scanned by a commercial, highly accurate terrestrial laser scanner, a handheld laser scanner, and smartphone-based scanning. Here, we wanted to compare our prototype against these devices and assess its performance.

In the following subchapters, we focus on the description of both experimental sites and how we carried out data collection across all devices, together with data processing and statistical evaluation. But first, we will describe the other state-of-the-art devices used for benchmarking.

### 3.1. Laser Scanners Used

*The RIEGL VZ-1000*

The RIEGL VZ-1000 (rieglusa.com) is a TLS capable of long-range data acquisition, with a maximum measurement range of up to 1400 m under favourable conditions. It uses echo digitisation and waveform processing to record multiple returns from a single laser pulse, which improves the reliability of measurements in structurally complex environments. With a data acquisition rate of up to 122,000 points per second, it is suitable for detailed three-dimensional mapping of difficult structures [10,30,31,32].

*The Stonex X120GO*

The Stonex X120GO (stonex.it) is an HMLS combining a FARO Focus X120 scanner with an automated rotating platform. The system enables 360° horizontal coverage by capturing multiple scans from a single position without manual repositioning. With a measurement range of up to 120 m and data acquisition rate of up to 976,000 points per second, it is well suited for rapid documentation of forest plots or individual tree structures, particularly in areas requiring efficient data collection with minimal user intervention [10,33,34].

*iPhone 15 Pro Max*

The SLD iPhone 15 Pro Max features a sensor that uses laser pulses to measure distances and create detailed 3D models of the environment. The scanner enables enhanced augmented reality experiences, improves the accuracy of applications using spatial mapping and facilitates the performance of imaging sensors. Apart from the specified operational range of approximately 5 m, Apple does not provide additional details about the accuracy or technical specifications of the LiDAR sensor used in its devices [35]. In environmentally oriented studies, similar SLDs were used for fast and user-friendly monitoring of forest parameters, although it provides lower accuracy in parameter estimation [9,21,24,36,37,38,39].

### 3.2. Lab Condition Experiment

**Figure 4:**
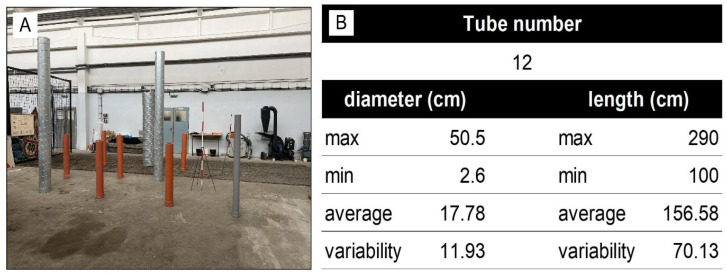
(**A**) Lab study site with the table of the reference tube parameters (**B**).

In the first experiment, we focused on comparing the devices in a controlled indoor environment in order to eliminate external influences, particularly weather conditions. The experiment was conducted in The Developmental Workshops and Laboratories of the Technical University in Zvolen. For the purposes of the experiment, 12 regular tubes of varying lengths and diameters were arranged in the laboratory, ensuring that they were spaced sufficiently apart (Figure 4). In this step, the evaluation of the proposed low-cost TLS system was conducted, focusing on its functionality, its ability to store data at the required quality, and the verification of the possibility of creating a complex point cloud.

The tubes were placed on a flat surface in an enclosed laboratory over an approximate area of 7 × 7 m. We chose tubes with different diameters in order to compare the impact of thickness on the error. The diameter of the tubes was measured using diameter tape at the height of 1 m. A total of 12 tubes were used in the experiment (Figure 4). The diameter of the tubes ranged from 2.6 cm to 50.5 cm, with an average diameter of 17.78 cm and a variability of 11.93 cm. The length of the tubes varied from 100 cm to 290 cm, with an average length of 156.58 cm and a variability of 70.13 cm (Figure 4B).

**Figure 5:**
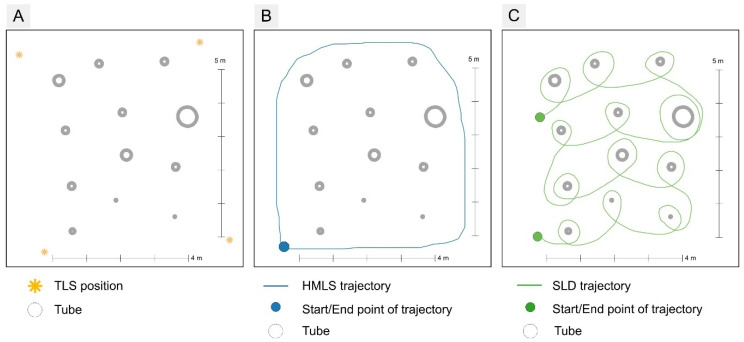
The lab condition experiment data acquisition schemes: (**A**) Scheme of TLSs: TLS Riegl and LCA-TLS (oriented to the centre of the lab study site) positioning, (**B**) trajectory of HMLS scanning, and (**C**) trajectory of SLD scanning within research plot.

As mentioned, we have used three types of laser scanners. When the TLS was used, including the prototype, we applied a multi-scan approach. The lab was scanned from its corner points, specifically from four positions (Figure 5A). For the HMLS, the lab plot was around the perimeter so that the scanner head was pointing inwards towards the lab plot (Figure 5B). For the SLD, the mobile 3D Scanner App by Laan Labs was used to perform the scan. For the SLD, each tube at the lab plot in shown in Figure 5C.

### 3.3. Experiment on Plantation of Fast-Growing Trees

**Figure 6:**
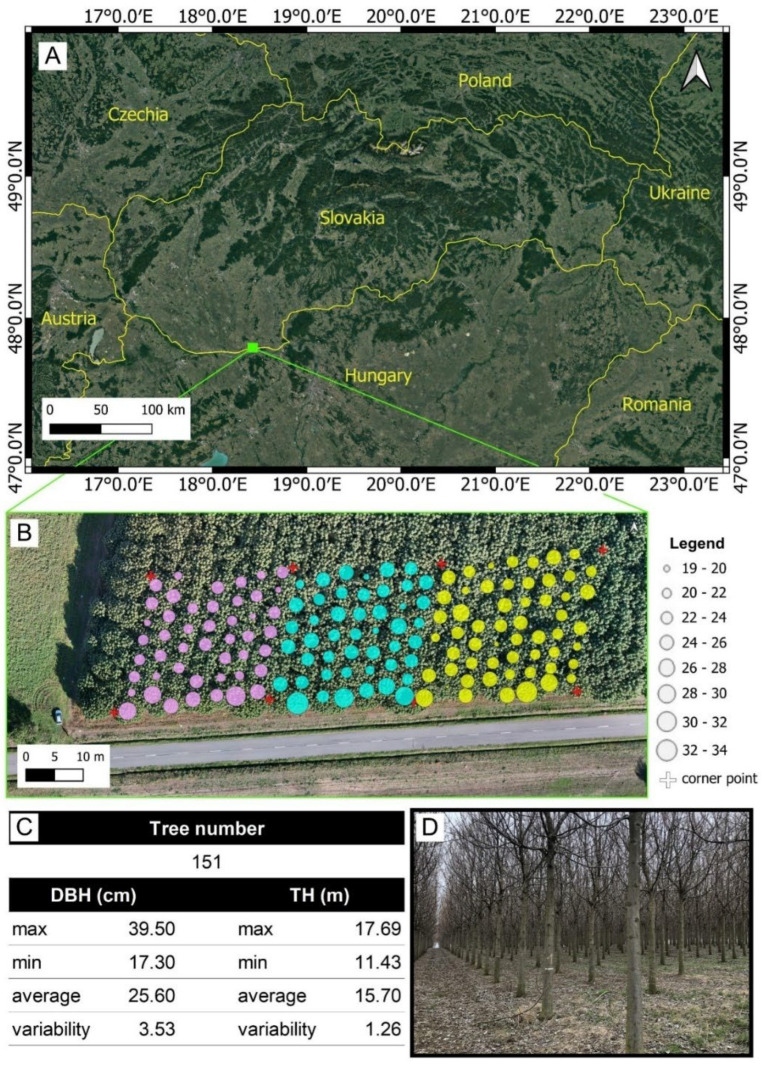
Plantation of Paulownia-Búč. The position of the site within Slovakia (**A**), together with a detailed orthomosaic of the Paulownia plantation (**B**). The circles represent the variation in DBH. On the bottom, detailed of within the plantation (**D**) and the table of the reference trees parameters (**C**). Different colors represent individual research plots.

After verifying the functionality, an experiment was conducted in a forest environment to assess its practical application. The second experiment was conducted in a Paulownia plantation located in south-west Slovakia (47.79° N, 18.45° E), in the Danube Lowland near the village of Búč (Figure 6). The plantation was established in spring 2015 (leaf-off season—Figure 2D) on flat terrain at an elevation of 108 m above sea level. A total of 200 container-grown seedlings of the hybrid Paulownia elongata × fortunei (Paulownia Cotevisa) were planted in rows with 4 × 4 m spacing (625 seedlings per hectare) [10].

For pilot testing of the TLS prototype, the Paulownia plantation provided optimal conditions, including uniform stem diameter, straight trunks free of lower branches, lower risk of occlusion, flat terrain, and the absence of understory vegetation. Altogether, 151 trees were considered and scanned within three connected plots. The whole scanned area had approximately 2700 m^2^. Based on the reference measurements, the average DBH was 25.6 cm, and the average TH was 15.7 m.

#### 3.3.1. Ground Truth Data Collection

Three research plots with dimensions 30 × 30 m were established (Figure 6B). Each plot’s corner point was secured on the terrain using plastic geodetic markers serving also as ground control points (GCPs). The positions of the GCPs were measured using a Topcon 9000 total station to ensure the permanent stabilization of the research plots in the field and to facilitate the handling of the resulting point clouds. The DBH of each tree within the plots was measured directly with a diameter tape at a height of 1.3 m. A calliper was not used, as the ellipsoidal shape of Paulownia trunks could reduce the accuracy of DBH measurements. The use of a diameter tape ensured reliable reference data. For TH evaluation, the reference data were measured using altimeter Haglöf Vertex 5. TH derived from all used devices was compared against this reference.

#### 3.3.2. Laser Scanning

**Figure 7:**
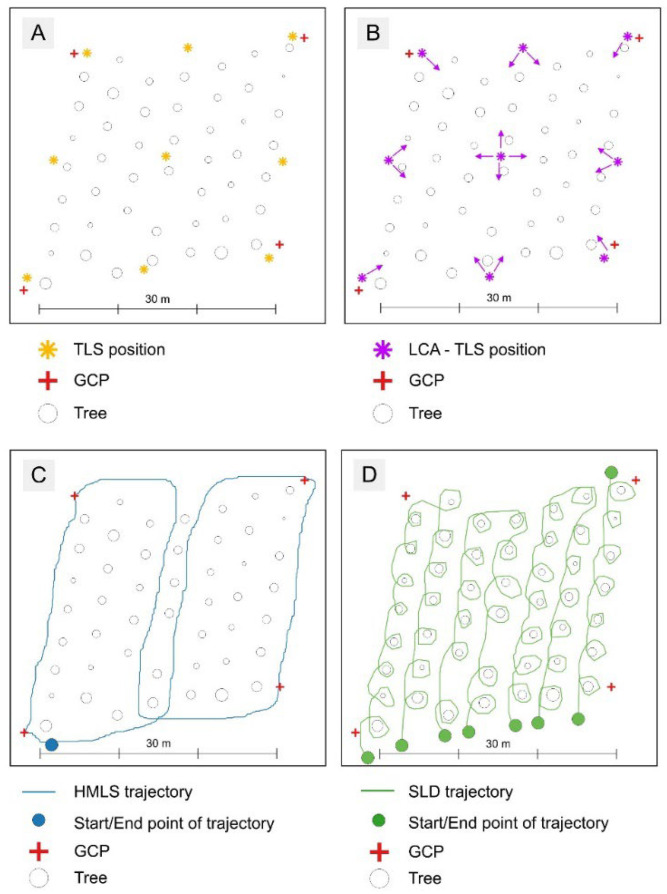
The Forest condition experiment data acquisition schemes: (**A**) Scheme of TLS positioning, (**B**) Scheme of LCA—TLS where the arrows indicate the rotation of the prototype during data acquisition, (**C**) trajectory of HMLS scanning, and (**D**) trajectory of SLD scanning within research plot.

The scanning with the LCA-TLS and the Riegl scanner followed the same multi-scan approach as in the lab experiment. To reduce occlusion, data were collected from nine different scanner positions per plot. Due to a narrower field of view, the total number of scans per plot was 16 for the LCA-TLS (Figure 7), instead of 9 for TLS (Riegl). In post-processing, the individual scans were registered and merged into a single unified point cloud per plot. The full scanning procedure for each plot, including changing the scanner position and setup, took approximately 21 min for the Riegl scanner and 13 min for LCA-TLS. For the HMLS survey, the operator carried the scanner by hand, walking both around the perimeter and through the centre of the plot, as shown in Figure 7B. Scanner initialization before every scan took approximately 1 min, while the scanning process itself lasted around 4 min. During SLD data acquisition, the scanning path was adapted to the iPhone LiDAR’s maximum range of 5 m. As shown in Figure 7C, the operator carried the device by hand and walked along the rows, passing by each tree. Due to memory limitations on the device and the complexity of the scanning process, it was not possible to capture the entire research plot in a single scan. Instead, only 1 to 2 rows were scanned at a time. The scanning process lasted around 8 min per plot.

### 3.4. Data Processing

#### 3.4.1. Post-Processing of Point Clouds

**Figure 8:**
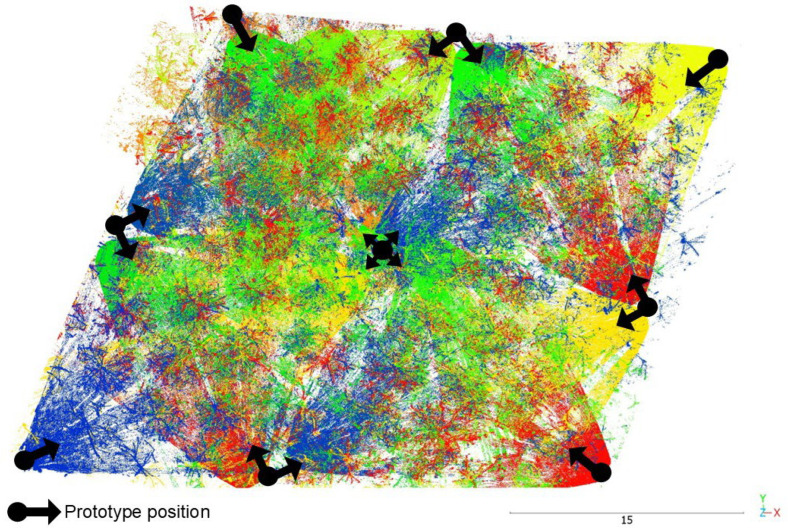
Visualization of processed LCA-TLS point cloud. Individual scans are colour-coded; Arrows indicate scanner orientation during data acquisition. Different colors are used to distinguish individual scans.

Data registration from LCA-TLS is performed using open-source software developed by the authors called Multi view TLS registration v. 0.72, initially designed for TLS data registration, available at https://github.com/MapsHD/HDMapping/releases/tag/v0.72 (accessed on 6 October 2025). It is extended for mobile mapping applications [40]. The processing pipeline is as follows:

- Data collection as multiple overlapping scans (Figure 8).
- Initial manual data alignment.
- Automatic consecutive data registration with ICP (Iterative Close Point).
- Multi view data registration with NDT (Norma Distribution Transform) [41].

To extract point clouds from the TLS and HMLS, the raw data were pre-processed using the software provided by the scanner manufacturers. Data from the RIEGL VZ-1000 were processed using RiSCAN PRO 2.1.2 software, while data from the Stonex X120 GO were pre-processed using GOpost software. For SLD scanning, this pre-processing step is not required, as the point cloud can be directly exported from the mobile device. Partial scans acquired with the SLD were merged into a single scan for each plot.

#### 3.4.2. DBH Extraction

**Figure 9:**
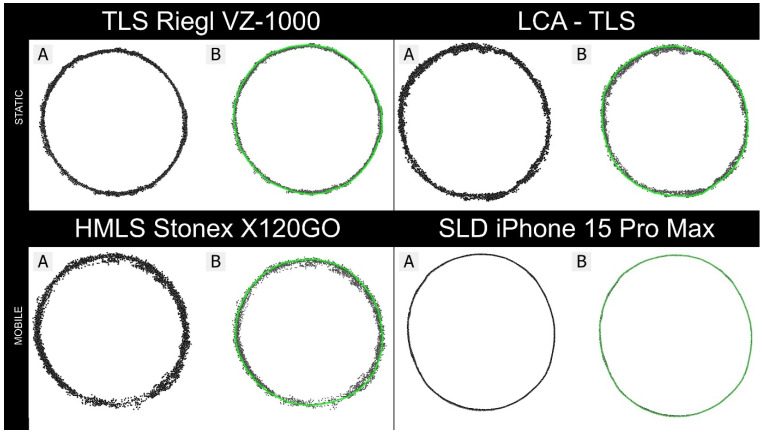
Sample cross-sections from all tested devices for DBH estimation: Cross-section (**A**) with the green outlining the polygon represents the perimeter (**B**) used for DBH calculation.

To estimate DBH, 2 cm thick cross-sections were created in CloudCompare (CloudCompare (version 2.12) [GPL software] (2021) retrieved from http://www.cloudcompare.org (accessed on 27 October 2025). To estimate DBH, the Cloth Simulation Filter (CSF) in CloudCompare [42] was applied twice to the same point cloud, first with a height threshold of 1.35 m and then with 1.30 m. The intersection of the two resulting ground-classified slices produced a 5 cm thick cross-section, from which DBH was measured. The cross-section was exported in .txt format and thus attached for further processing-DBH estimation from perimeter in QGIS 3.16.3., where the tree perimeter was manually measured and converted to DBH. To compare the accuracy of the devices, we decided to estimate the parameters manually, because using software could introduce an error in DBH estimation rather than an error attributable to the device itself. To estimate the DBH, a polygon accurately outlining the outer perimeter was manually created for each cross-section, and its length was then recalculated to determine the diameter in the subsequent step (Figure 9). The figure shows a polygon outlining the outer perimeter of tubes in cross-sections of point clouds captured by the tested devices.

#### 3.4.3. TH Extraction

**Figure 10:**
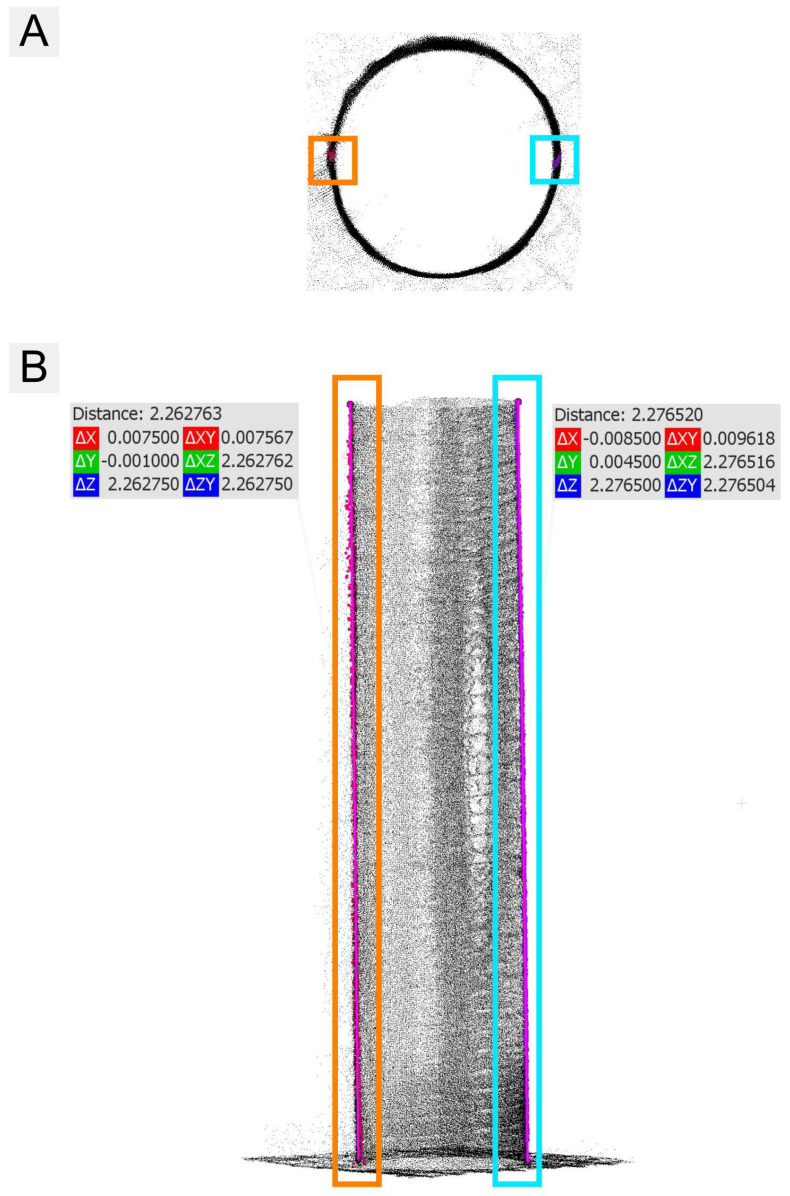
The lab condition point cloud-based TH estimation: Vertical segments of the point cloud visualized from top (**A**) and side view (**B**). Different colors are used to indicate the position of the measurement segments (left and right). The pink line indicates the measured height.

For TH estimation in lab conditions, two vertical segments of the point cloud in line with the z-axis were created on each tube in CloudCompare (Figure 10A). The Point picking tool was used to measure the distances between the points at the ends of the segments. The distances represented the lengths of the tubes (Figure 10B).

**Figure 11:**
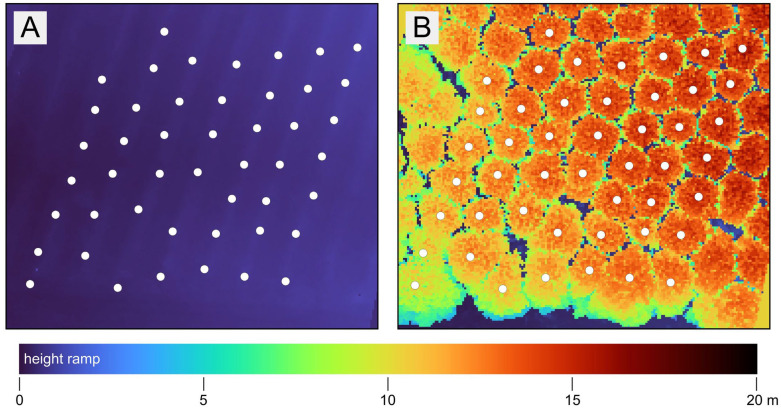
The forest point cloud-based TH estimation: DTM (**A**) and nDSM (**B**) with tree positions represented by white points.

To estimate TH in the forest conditions, a digital terrain model (DTM) (raster layer of minimum values) was first created from each point cloud (Figure 11A), followed by a digital surface model (DSM) (raster layer of maximum values). These two layers were then subtracted (Figure 11B) from each other to produce a normalized Digital Surface Model (nDSM = DSM − DTM) [43].

Based on this raster, the value corresponding to the cell that overlapped with the centroid of the stem was extracted for each position of the stem. This process made it possible to obtain height values for each technology, with the exception of the SDL, where this was not possible due to limited sensor range. In QGIS, the Raster Calculator module was used to create a nDSM and tree height extraction via the Sample Raster Value module.

### 3.5. Point Cloud Quality Evaluation

The cross-sections of tree stems were extracted from 3D point clouds and subsequently transformed from Cartesian coordinates (X, Y, Z) into polar coordinates, where the angle θ represents the azimuth of the point relative to the stem centre, and the radial distance r corresponds to its radius. The cross-section centre and reference radius were determined using analytical circle fitting. For subsequent analysis, the cross-section was “unwrapped” into a two-dimensional form, where the X-axis represents the angle θ (0–360°) and the Y-axis represents the deviation of individual points from the reference circle (in millimetres). To model the ideal stem outline, Fourier approximation with a fixed number of harmonic terms was used, enabling the capture of periodic shape variations while suppressing high-frequency noise. The Fourier method was chosen over local smoothing approaches (e.g., LOESS [44]) because it provides a global, periodic, and analytically defined model of the perimeter, ensuring continuity and periodicity over the 0–360° range and eliminating the risk of edges or discontinuities at the angular boundaries.

The noise was quantified based on residuals, i.e., the differences between the measured values and those predicted by the Fourier function. As an indicator, the interval containing 95% of all residuals was used, with its width (in mm) representing the range in which the majority of cross-section points lie after the removal of systematic shape deviations. A smaller interval width indicates a lower level of noise and higher measurement accuracy, whereas a larger width reflects a higher degree of random deviations, typical for devices with greater sensor noise.

### 3.6. Statistical Evaluation

Estimation errors were obtained as the difference between the reference and estimated values (1, 2).

(1) $${DBH}_{err}={DBH}_{est}-{DBH}_{r}$$

where *DBH_err_* is a calculated error of estimated DBH. *DBH_est_* is estimated from the point cloud and *DBH_r_* represents values measured in the field/laboratory conditions.

(2) $${TH}_{err}={TH}_{est}-{TH}_{r}$$

where *TH_err_* is the calculated error of the estimated *TH*. *TH_est_* is estimated from the point cloud, and *TH_r_* is the manually measured *TH*.

(3) $${RMSE}_{DBH}=\sqrt{\frac{\sum_{i=1}^{n}({DBH}_{r}-{{DBH}_{est})}^{2}}{n}}$$

(4) $${RMSE}_{TH}=\sqrt{\frac{\sum_{i=1}^{n}({TH}_{r}-{TH}_{est}{)}^{2}}{n}}$$

where *DBH_r_* and *TH_r_* represent measured values, ${DBH}_{est}$ and ${TH}_{est}$ represent estimated values, and *n* is the number of trees in the dataset.

A paired *t*-test was conducted to assess whether there were statistically significant differences between the estimated measurements and the reference values. This analysis was performed using the R statistical environment (R Core Team, 2024). To evaluate differences among devices in estimating *TH* and *DBH*, a one-way analysis of variance (ANOVA) was applied. When significant differences were detected, Tukey’s Honest Significant Difference (HSD) post hoc test was used to determine which devices differed significantly from each other.

A short questionnaire was developed to collect user-based evaluations of selected LiDAR devices with respect to their usability and efficiency during data collection and post-processing. Respondents were asked to rate each device using a 5-point scale. The questionnaire was distributed among 10 colleagues with practical experience in the use of these technologies. Overall, the questionnaire was completed by respondents who had interacted with all the technologies used in this experiment.

## 4. Results

### 4.1. Point Cloud Quality Evaluation: Distribution and Density

**Figure 12:**
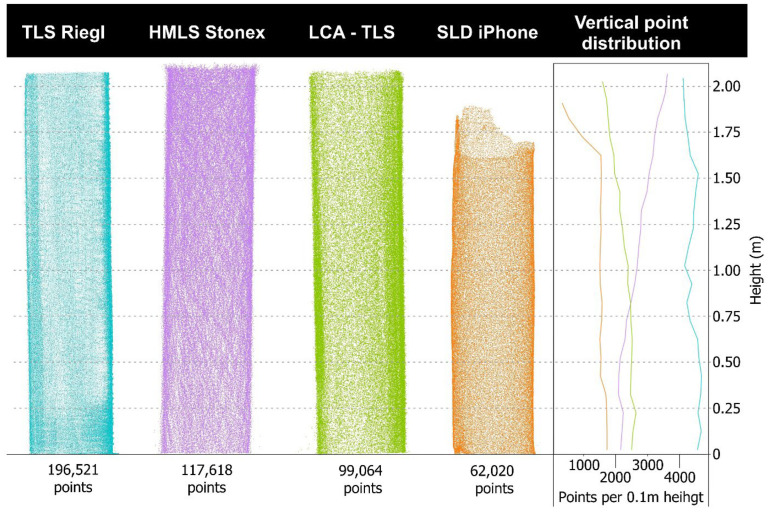
An illustration of the vertical point distribution of TLS, HMLS, LCA-TLS and SLD point clouds of an individual tube. An example is the tube ID10. Different colors represent individual devices: blue—TLS, purple—HMLS, green—LCA-TLS, and orange—SLD.

To focus on the geometric scanning performance of each device, measurements were conducted on cylindrical targets under controlled indoor conditions, without external influences such as wind, vegetation, or uneven ground. To assess point cloud quality, we selected a cylindrical target (ID10; Table 2) as a representative object, with the point cloud slice positioned at a height of 1.3 m, for detailed analysis under controlled indoor conditions (Figure 12).

Despite the uniform shape of the objects, the resulting point clouds varied notably among the four tested platforms in both density and spatial precision (Figure 12). LCA-TLS prototype captures about half as many points as conventional TLS Riegl (98,064 vs. 196,521 points) and roughly 20% fewer points than HMLS (117,618 points) and around 1.6 times more points than the iPhone (62,020 points). The density of LCA-TLS offers a stable yet lower density when compared to TLS and MLS, however, at a level that is sufficient for detailed reconstruction.

The unwrapped cross-sections (Appendix A.9) further clarify these differences: the iPhone produced the narrowest 95% residual band (6.79 mm; 958 points), indicating low random noise within the evaluated slice, albeit at the lowest density. The TLS Riegl yielded the densest dataset (2798 points) with a moderate residual band (14.17 mm), but its residuals contain a relatively large random component. HMLS exhibited the widest residual band (25.47 mm; 1998 points), while the LCA-TLS prototype showed intermediate density with a relatively wide band (22.57 mm; 1507 points), suggesting a mixture of random and systematic deviations. In summary, while Riegl excels in point density and continuous coverage of the cylindrical surface, the iPhone achieves the lowest random variability in the cross-section at the cost of reduced density; HMLS and LCA-TLS display broader residuals indicative of higher noise and/or systematic contour bias (Appendix A.9).

**Figure 13:**
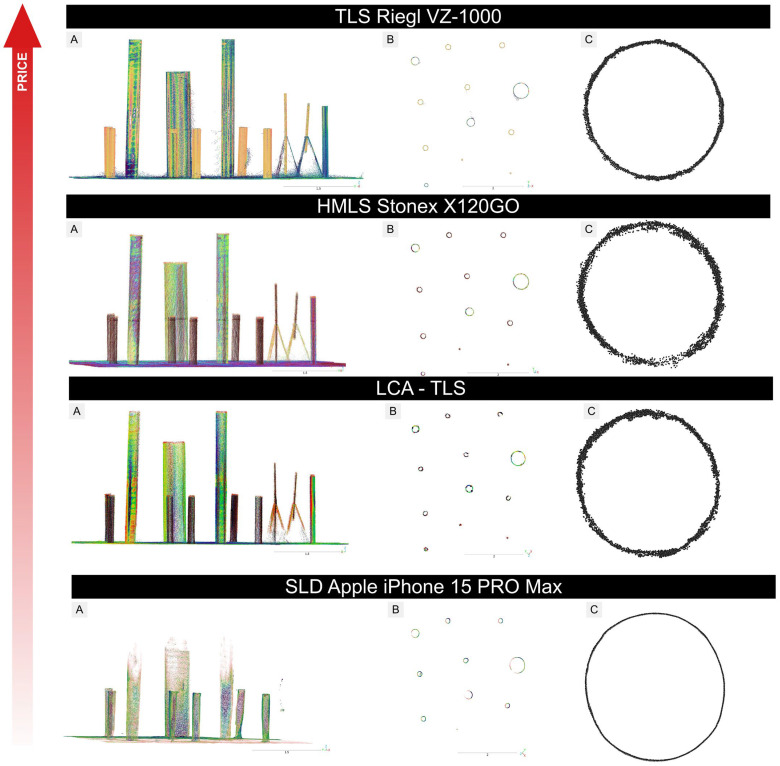
Comparison of the quality of point clouds obtained by the four tested devices under laboratory conditions, using objects of known geometry simulating tree stems. The visualizations include a side view (**A**), a cross-section view (**B**), and a cross-section at 1.3 m height representing the DBH level (**C**).

Visual interpretation of the point clouds (Figure 13) showed that all devices reproduced the cylindrical target with recognizable geometry, but with varying contour definition and completeness. The TLS Riegl preserved sharp edges and continuous point coverage along the surface, while the LCA-TLS prototype maintained overall shape with slightly reduced edge sharpness. The HMLS Stonex displayed minor contour irregularities and localized gaps, whereas the iPhone reconstruction appeared smooth and exhibited more uniform edges. These qualitative differences correspond with the cross-section profiles in Appendix A.9.

**Figure 14:**
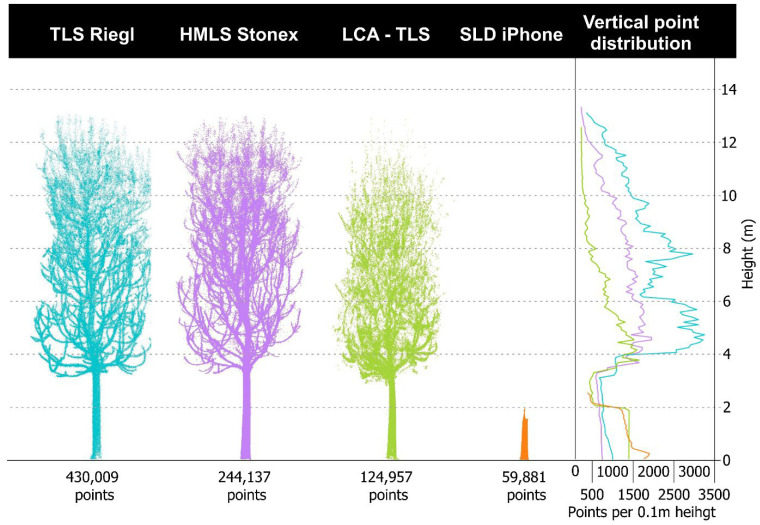
An illustration of the vertical point distribution of TLS, HMLS, LCA-TLS and iPhone point clouds of an individual tree. Different colors represent individual devices: blue—TLS, purple—HMLS, green—LCA-TLS, and orange—SLD.

When comparing the point clouds of trees (Figure 14), clear differences emerge among the technologies. Based on the average number of points per tree point cloud, LCA-TLS provides about half as many points as HMLS (124,957 vs. 244,137) and only around 29% of the points captured by TLS (430,009), yet it still achieves roughly double the points compared to the iPhone (59,881). While LCA-TLS represents a low-cost option with moderate point density and stable performance, it falls short of the level of detail achievable with conventional TLS or HMLS. The TLS Riegl produced a dense and complete point cloud, capturing both crown and stem structures clearly. HMLS, with the highest point count, also maintains a consistent and detailed scan of the trunk and upper canopy.

In terms of vertical point distribution, TLS and HMLS maintain high and consistent densities throughout the tree height. LCA-TLS provides a stable but lower density up to around 8–10 m, with a significant drop above this height. The iPhone records almost no points beyond 2 m, resulting in poor coverage of the upper canopy.

**Figure 15:**
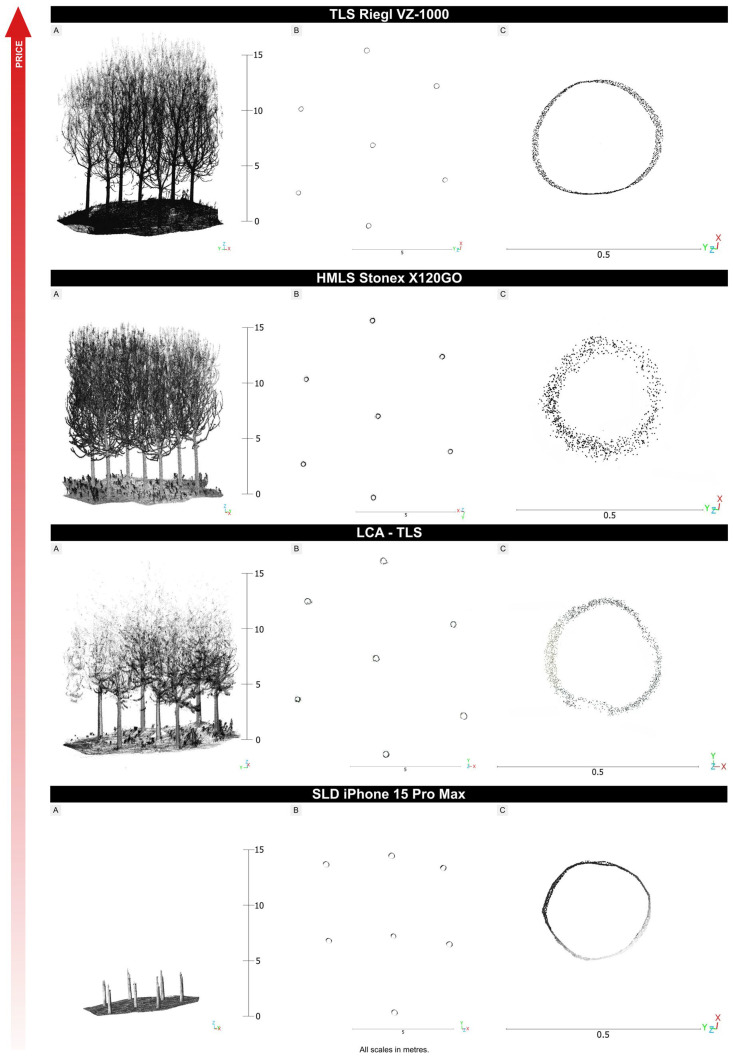
Comparison of the quality of point clouds generated by four tested devices under fast-growing tree plantation conditions. Three views are shown: side view of the stand (**A**), top cross-section view (**B**), and a cross-section at breast height (**C**). The devices are arranged from top to bottom in order of increasing cost (indicated by the arrow).

The cross-sectional view shows a regular and complete outline of the stems with TLS (Figure 15). For the evaluation we used a representative specimen from our dataset, with the point-cloud cross-section positioned at 1.3 m above ground. The HMLS Stonex captured individual stems with sufficient clarity, although the point density was lower. The stem outlines in the cross-section are preserved but appear less continuous. The LCA-TLS achieved a lower point density, especially in the crown area, but the stem shapes remain recognizable. The SLD iPhone captured only the lower parts of the stems. The point density is significantly lower, and the upper parts of the trees are missing. The cross-section is less detailed, but the stem outlines are still discernible.

The unwrapped cross-sections of the stem (Appendix A.9) quantify these differences at DBH: the iPhone yielded the narrowest 95% residual band (8.58 mm; 1166 points), TLS Riegl showed a moderate band (17.42 mm; 425 points), LCA-TLS a wider band (37.85 mm; 630 points), and HMLS the widest band (49.68 mm; 511 points). Thus, at DBH, the iPhone exhibits the lowest random variability within the cross-section (albeit with limited overall tree coverage) and TLS combines moderate variability with dense, continuous scanning, while HMLS and LCA-TLS display broader residuals indicative of higher noise.

### 4.2. Lab Condition Experiment

In this phase of the experiment, all tested technologies achieved a 100% object detection rate, except for the LiDAR sensor integrated in the iPhone. This device failed to detect two low-diameter tubes, resulting in a detection rate of 83.3% (Table 2). These undetected objects were excluded from the calculation of RMSE and bias, as the iPhone provided no diameter estimates for them. This approach reflects standard practice in quantitative evaluations, where only successfully detected targets are considered when calculating metrics, thereby separating measurement performance from detection capability. Regarding DBH estimation accuracy, the TLS Riegl performed as expected, achieving the lowest deviation with an RMSE of 0.62 cm. Interestingly, the prototype based on the Livox Avia sensor outperformed both commercially available HMLS solutions, iPhone LiDAR and Stonex. With an RMSE of 1.23 cm and a bias of 1.03 cm, it ranked just behind the professional TLS, demonstrating its potential as an effective and affordable alternative (Table 2).

When evaluating the accuracy of tree height estimation, the TLS Riegl again proved to be the most precise, achieving the lowest RMSE of 2.41 cm and a bias of 2.19 cm, following the LCA-TLS, with an RMSE of 5.21 cm, confirming its consistently solid performance. The HMLS Stonex reaching an RMSE of 7.36 cm, while the iPhone showed significantly lower accuracy, with an RMSE of 38.02 cm and a substantial negative bias (−16.06 cm) (Table 3). As the iPhone failed to detect two objects during the measurement process, these undetected cases were excluded from the RMSE and bias calculations to ensure a consistent and objective comparison among devices.

### 4.3. Forest Condition Experiment

#### 4.3.1. TDR

Compared to laboratory conditions, slightly lower precision values were observed in the plantation, which is expected due to the increased variability of environmental conditions. Across all test sites, the sum of trees is 151 and all devices achieved a 100% tree detection rate (TDR), confirming their reliability in identifying tree stems under these conditions.

#### 4.3.2. DBH

The most accurate DBH estimates were obtained using the TLS Riegl system (Table 4), which achieved the highest coefficient of determination (R^2^ = 0.973) and the lowest RMSE value (0.754 cm). These results align with expectations and confirm the system’s reliability under field conditions. The HMLS Stonex system followed, with R^2^ = 0.944 and RMSE of 1.317 cm. The low-cost LCA-TLS prototype demonstrated strong performance as well, ranking third in overall accuracy with a RMSE of 1.503 cm and R^2^ = 0.943. The iPhone exhibited the highest RMSE among all devices (2.007 cm), indicating the lowest accuracy.

**Figure 16:**
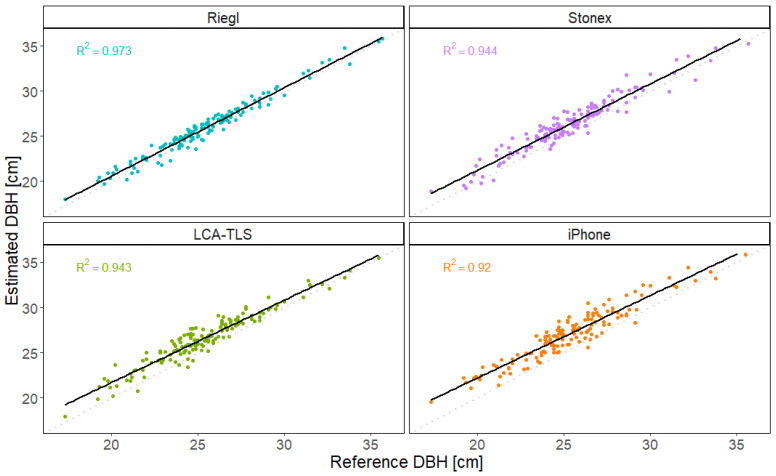
Conventional and point cloud-based methods for DBH measurement, according to each device used, with its regression line and r squared.

Beyond differences in overall accuracy, the dispersion of DBH estimates varied noticeably among devices. The Riegl scanner produced measurements tightly clustered around the regression line, indicating high consistency. Both Stonex and LCA-TLS showed slightly greater spread, particularly in mid-range diameters. The iPhone data exhibited the widest scatter, suggesting higher variability and less reliable measurements, especially for smaller trees (Figure 16).

**Figure 17:**
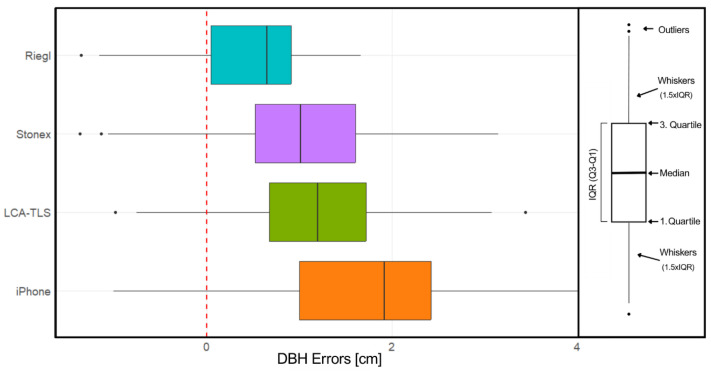
Boxplot of DBH Estimation Errors by Device in Forest Conditions, where the central line represents the median, the edges of the box indicate the first (Q1) and third quartiles (Q3), and the whiskers extend to the most extreme data points within 1.5 times the interquartile range (IQR) from the quartiles. Data points beyond the whiskers are considered outliers and are plotted individually as dots. Different colors represent individual devices: blue—TLS, purple—HMLS, green—LCA-TLS, and orange—SLD.

The Riegl exhibited the lowest variance and mean error values (Figure 17), with a median error of 0.649 cm, indicating minimal bias and high measurement accuracy. The interquartile range (IQR) was approximately 0.869 cm, spanning from 0.044 to 0.913 cm, while the whiskers extended from −1.155 cm to 1.662 cm. This distribution suggests slight underestimation in some instances but overall consistent performance. The Stonex showed a moderately higher median error of 1.012 cm and a wider error distribution, with an IQR of approximately 1.088 cm and whiskers ranging from −1.060 cm to 3.146 cm. These values indicate a tendency toward slight overestimation and greater variability compared to Riegl. The LCA-TLS prototype presented a further increased median error of 1.201 cm, accompanied by an IQR of approximately 1.041 cm and whiskers spanning from −0.755 cm to 3.080 cm, reflecting overestimation while maintaining a precision level comparable to that of Stonex. Despite these limitations, the LCA-TLS prototype still provided usable estimates. The iPhone demonstrated the highest median error of 1.920 cm and the greatest variability, with an IQR of approximately 1.423 cm and whiskers ranging from −1.007 cm to 4.110 cm. This distribution highlights significant overestimation and the lowest reliability among the devices tested for DBH measurement.

The ANOVA revealed a statistically significant difference between the devices used for DBH estimation (Appendix A.1). Pairwise comparisons were then conducted using the Tukey HSD post hoc test to identify specific differences between devices (Appendix A.2).

**Figure 18:**
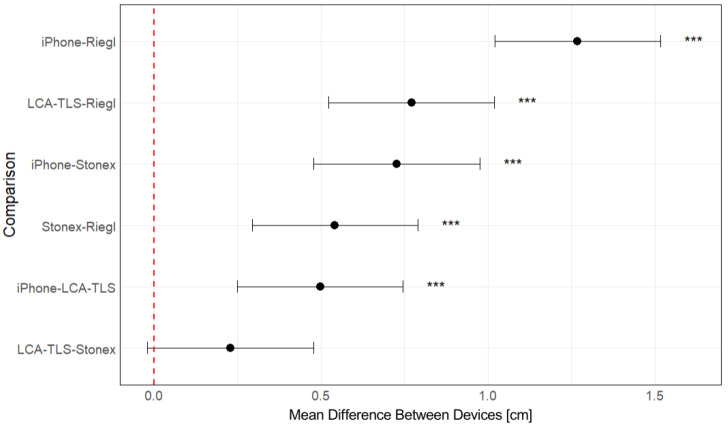
Tukey HSD: Pairwise Differences Between Devices. Results of the post hoc Tukey HSD test comparing the mean differences in DBH estimates between device pairs. The red dashed line represents the zero-difference threshold, indicating no statistically significant difference in DBH estimates. Confidence intervals that do not cross this line indicate significant differences between device pairs. Significance levels are denoted as follows: *** *p* < 0.001.

The results of the Tukey HSD test (Figure 18) confirmed statistically significant differences in DBH estimation accuracy between most of the evaluated devices. The iPhone consistently produced the highest DBH values, showing significant overestimation compared to all other devices. The low-cost LCA-TLS prototype also differed significantly from Riegl (*p* < 0.001), which exhibited the smallest mean difference and highest accuracy among the devices; however, the mean deviation of the LCA-TLS was smaller than that of the iPhone. Importantly, no statistically significant difference was found between the LCA-TLS prototype and the HMLS Stonex, indicating comparable accuracy between the prototype and this commercial HMLS system.

The analysis of the mean differences in measurements of the individual devices compared to the reference value using a paired *t*-test (Appendix A.3 and Appendix A.4) showed statistically significant deviations in all cases (*p* < 0.001). The Riegl device exhibited the smallest mean difference (0.470 cm), indicating the highest accuracy relative to the reference. The Stonex and LCA-TLS devices showed mean differences of 1.012 cm and 1.241 cm, respectively, thus being less accurate than Riegl but still relatively consistent. The largest deviation was recorded by the iPhone, with a mean difference of approximately 1.739 cm. The confidence intervals for all devices did not cross zero, confirming the statistical significance of the differences.

#### 4.3.3. Tree Height

When evaluating tree height, the iPhone 15 Pro Max was not included in this analysis, as the limited range of its built-in LiDAR sensor did not allow for reliable capture of the upper parts of the trees. In the comparison of estimated TH (Table 5), the TLS Riegl system achieved RMSE 0.290 m, the HMLS Stonex device achieved an RMSE of 0.468 m, whereas the LCA-TLS prototype exhibited a higher RMSE of 0.992 m.

**Figure 19:**
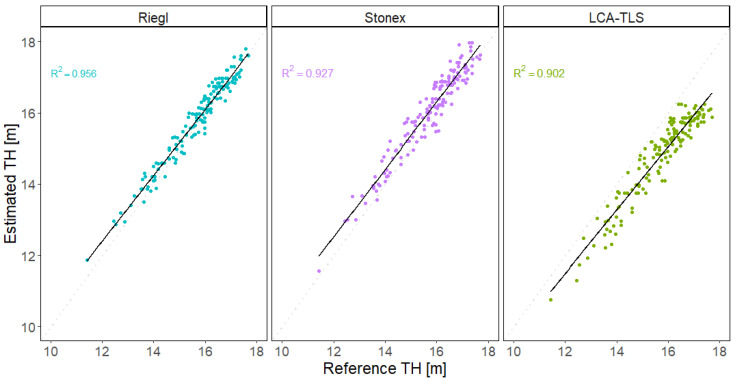
TH measurement, according to each device used, with its regression line and r squared.

All devices showed a strong correlation between reference and estimated TH values, with the Riegl system achieving the closest fit to the regression line (Figure 19). HMLS Stonex and LCA-TLS followed with similar results, with the LCA-TLS prototype showing a slightly larger dispersion of estimates, especially at lower height values. The LCA-TLS prototype showed the lowest accuracy and the greatest variability.

**Figure 20:**
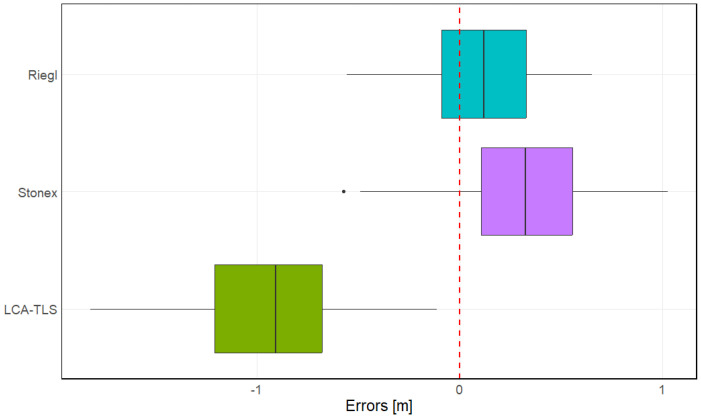
Boxplot of TH Estimation Errors by Device in Forest Conditions, where the central line represents the median, the edges of the box indicate the first (Q1) and third quartiles (Q3), and the whiskers extend to the most extreme data points within 1.5 times the interquartile range (IQR) from the quartiles. Data points beyond the whiskers are considered outliers and are plotted individually. Different colors represent individual devices: blue—TLS, purple—HMLS, green—LCA-TLS.

The comparison of TH measurement errors revealed distinct performance characteristics across the evaluated devices (Figure 20). The LCA-TLS prototype exhibited the largest negative median error of −0.907 m, indicating a tendency to underestimate estimation of TH. The interquartile range (IQR) was approximately 0.529 m, spanning from −1.209 m (Q1) to −0.680 m (Q3), with whiskers extending from −1.819 m to −0.112 m. This relatively narrow dispersion suggests consistent measurement performance, although with a pronounced tendency toward underestimation of tree height. In contrast, the Stonex displayed a positive median error of 0.324 m, suggesting a slight overestimation of tree height. Its IQR of approximately 0.448 m, ranging from 0.106 m to 0.554 m, indicates moderate variability, and whiskers extended from −0.492 m to 1.026 m. One mild outlier (−0.574 m) was also detected, indicating occasional underestimation. The Riegl showed the lowest median error of 0.119 m and the narrowest IQR (approximately 0.415 m), ranging from −0.089 m to 0.326 m. The whiskers extended from 0.558 m to 0.650 m, suggesting high accuracy and low variability in tree height measurements. Among all devices, Riegl demonstrated the most consistent and accurate performance for TH, while LCA-TLS tended to underestimate and Stonex showed a moderate overestimation bias.

Analysis of variance (Appendix A.5) for the variable TH showed that the choice of device had a highly significant effect on the measured values (F = 591.9, *p* < 0.001). The model explained a substantial portion of the variability, with between-device variance markedly exceeding within-group variance.

**Figure 21:**
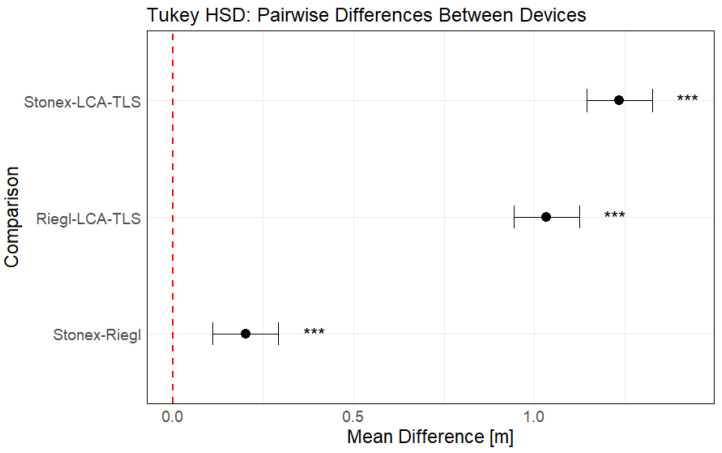
Tukey HSD: Pairwise Differences Between Devices. Results of the post hoc Tukey HSD test comparing the mean differences in TH estimates between device pairs with vertical lines indicating 95% confidence intervals (CI). The red dashed line represents a zero-difference threshold, indicating statistical equivalence in TH estimates between devices. Significance levels are denoted as follows: *** *p* < 0.001.

Subsequent post hoc analysis using Tukey’s test revealed statistically significant differences among all three device pairs (Appendix A.6; Figure 21). The Stonex HMLS showed, on average, 0.201 m higher error values than the Riegl (*p* < 0.001). The LCA-TLS prototype underestimated tree height in comparison to both professional systems, with a mean difference of −1.033 m relative to Riegl (*p* < 0.001) and −1.235 m relative to Stonex (*p* < 0.001).

Statistical comparison of tree height estimates using paired *t*-tests (Appendix A.7 and Appendix A.8) revealed significant differences between all tested devices and the reference values. The Riegl showed a small but statistically significant mean overestimation of 0.122 m (95% CI: 0.079 to 0.164 m, *p* < 0.001). The Stonex device exhibited a greater mean overestimation of 0.323 m (95% CI: 0.269 to 0.378 m, *p* < 0.001), indicating a consistent upward bias. In contrast, the LCA-TLS prototype significantly underestimated tree height, with a mean difference of −0.912 m (95% CI: −0.974 to −0.849 m, *p* < 0.001). These findings confirm that all devices deviated significantly from the reference measurements, with the direction and magnitude of error varying by device.

### 4.4. Time Requirements, Cost and Ease of Use/Overall Evaluation

A summary of the overall evaluation is in Table 6. The cost evaluation considered the prices of both hardware and software necessary to generate point clouds suitable for extracting selected dendrometric parameters. Pricing information was obtained either from official distributor quotations or purchase invoices provided by individual manufacturers. It should be noted actual prices may vary by country, depending on the availability and procurement conditions of local distributors. Currently, the cost of alternative TLS devices from Riegl, such as the VZ-400i or VZ-600i models, ranges from €90,000 to €130,000, including proprietary data processing software. The Stonex X120GO MLS scanner, along with the required software, is priced in the range of €30,000 to €35,000. The prototype was constructed at a total cost of €2050. In contrast, the iPhone 15 Pro Max, which also features an integrated LiDAR sensor, is available at a considerably lower price, ranging from approximately €850 to €1200. The processing software is freely available.

For TLS, data acquisition on a single research plot using nine scanning positions required approximately 21 min. Processing the raw data and aligning the individual scans into a unified point cloud using RiSCAN Pro software took an additional 26 min. In the case of HMLS, data collection per plot was completed in less than 5 min, while subsequent processing using the GOpost software required approximately 20 min. Low-cost TLS data acquisition using 16 scanning positions required approximately 40 min and data processing 49 min. For the SLD system, data collection time was also relatively short, taking less than 8 min per plot. As the iPhone generates point clouds in real time, no post-processing was necessary.

In addition to time efficiency, the overall user accessibility of each device was evaluated. The values in Table 6 represent the average score calculated from the total points assigned by respondents to each device in two categories: data collection and post-processing. The highest user accessibility rating was achieved by the TLS Riegl VZ-1000 (3.5), followed by the low-cost LCA-TLS (2.6) and HMLS Stonex X120GO (2.4). The lowest score (1.3) was assigned to the SLD iPhone 15 Pro Max, suggesting limited practical usability compared to the other devices.

## 5. Discussion

### 5.1. From Metrics to Insights: Comparing a Low-Cost TLS with the State- of the Art in Terrestrial Point Cloud Sources

All measurements were conducted under identical conditions at the same location, with consistent object placement, and with maximum possible elimination of environmental variability. The differences in accuracy across the tested devices can be attributed only to the technical and methodological aspects of each solution. Overall, the LCA-TLS offers a solid compromise between DBH accuracy, cost-efficiency, and technical simplicity, and in the case of TH, lower reliability must be expected. This limitation appears to be shared across the most terrestrial devices. To form a complete picture, it is necessary to consider not only accuracy but also biases and consistency across devices.

The results of paired *t*-tests confirmed the presence of statistically significant deviations between each device and the reference for both analyzed variables (DBH and TH). All tested devices showed consistent directional biases, either overestimation or underestimation of tree height and diameter. These shifts highlight the need to critically assess absolute accuracy when using such technologies for inventory or monitoring purposes. Despite the growing interest in the use of new and affordable technologies for forest data collection, there is a notable lack of current studies directly addressing low-cost TLS systems. While there are several articles on low-cost solutions, most of them deal with mobile systems [23,29,45,46] and specific experiments with sensors in SLDs [21,35,39,47] which is not the same as a low-cost TLS system.

In addition, many of the systems labelled as “low-cost” in these studies still cost several tens of thousands of euros. While this may be reasonable compared to high-end TLS systems, it does not reflect what can truly be considered a low-cost solution for practical forest data collection. Therefore, evaluating real affordability requires reassessing what “low-cost” actually means in laser scanning contexts.

This gap in the literature, along with the methodological advantages of static scanning, motivated the development and testing of our low-cost TLS solution. Before presenting its performance, we compare it with relevant alternatives based on key forest inventory parameters. Although HMLS systems offer high flexibility, efficient spatial data collection, and better affordability compared to standard TLS, for certain types of tasks, a TLS-based solution remains a more methodologically appropriate choice. Importantly, TLS enables targeted, controlled, and repeatable scanning, which improves coverage without introducing additional noise, even as point density increases. In contrast, mobile systems, due to their motion, may blur the details of small or complex objects. Moreover, when a standardized and repeatable methodology is required for long-term monitoring, TLS proves advantageous due to its precisely defined scanner positions and consistent scanning conditions. In this context, we have designed and tested a low-cost TLS system that represents a significantly more affordable alternative to commercial TLS solutions, without a major compromise in output accuracy for DBH estimation. Although static solutions may be perceived as less flexible compared to mobile systems, the higher accuracy of the LCA-TLS in DBH estimation and its substantially lower cost make it a highly practical option for specific tasks.

Compared to other low-cost methods for creating point cloud close-range photogrammetry (CRP), the LCA-TLS is an active device that enables easy and direct measurements. In contrast, the CRP is highly sensitive to outdoor lighting conditions, making it difficult to use in forest environments [48]. This is a key disadvantage compared to LiDAR-based systems, where data acquisition is generally more robust. Moreover, accurate data collection is crucial, and the post-processing workflow in CRP can be demanding, especially for users unfamiliar with the methodology.

*Tree detection rate*

Comparing the LCA-TLS to the low-cost MLS LC-MLS [49], which represents a true low-cost mobile solution without SLAM, the LCA-TLS prototype demonstrated comparable or even higher accuracy in DBH estimation (RMSE = 1.503 cm, bias 1.241, rRMSE = 5.872%) at a substantially lower construction cost (~€2050). The Balestra’s LC-MLS system [49] achieved a DBH RMSE of 2.20 cm and a bias of +1.62 cm (rRMSE = 6.11%), with a reported cost of approximately €7000.

Similar in design to our experiment, the low-cost TLS system BEE from Beijing Forestry University, which uses a two-dimensional laser scanning sensor (SICK LMS-511) mounted on a rotating platform, was tested in a mixed plantation. In our study, the LCA-TLS achieved a 100% TDR in a stand with a density of 562 trees/ha. In comparison, the BEE prototype [48] reached TDRs ranging from 86.36% to 100% in plots with stem densities of 1400 to 2200 trees/ha. Interestingly, the 100% TDR was not achieved in the lowest-density plot.

*Diameter at the breast height*

For DBH estimation, all tested devices showed statistically significant overestimation compared to manually measured reference data. The LCA-TLS prototype overestimated DBH by an average of 1.241 cm, representing the second highest bias among the evaluated devices. The Riegl achieved the lowest error, with a mean bias of 0.470 cm, although this difference was still statistically significant. Stonex showed a mean overestimation of 1.012 cm, while the iPhone recorded the highest bias at 1.739 cm (Table 4).

The deviations in DBH estimation observed in this study are consistent with the other findings [49]. Although the LCA-TLS prototype is a static terrestrial scanner, its simple design, good mobility, and low-cost place it among affordable systems comparable to low-cost MLS devices. Its average overestimation of 1.24 cm is close to the +1.62 cm reported by Balestra et al. [49] for the LC-MLS. An even closer match was observed with the Stonex device, which showed a bias of +1.01 cm, almost identical to the +1.02 cm reported for the GeoSLAM ZEB Revo RT. Both of these systems are commercially available mobile scanners commonly used in professional practice and are considerably more expensive than our prototype.

Our results also align with those of P. Wang et al. (2019), who presented the BEE scanner, a low-cost terrestrial laser scanner based on the SICK LMS-511, with a total cost under 10,000 USD. Their system showed a positive bias of +0.64 cm (RMSE = 1.27 cm) for DBH estimation, which is lower than our result for the LCA-TLS (+1.24 cm), but still within a comparable accuracy range. The similar direction of error (DBH overestimation) observed in both studies suggests that this trend is consistently present across different types of low-cost TLS systems. In terms of DBH estimation, the BEE system reached an RMSE of 1.270 cm, which is slightly better than the 1.503 cm achieved with LCA-TLS. This difference may be due to the Livox Avia sensor used in our system, which can have lower point density in specific horizontal slices as also indicated by our DBH cross-section analysis (Appendix A.9), where LCA-TLS captured fewer points and showed a wider residual band compared to TLS Riegl. Since it generates a non-uniform point pattern using a spiral scanning method, the number of points captured at height of 1.3 m may be lower or unevenly distributed (Avia LiDAR sensor-Livox). In contrast, the SICK LMS-511 implemented in BEE is a 2D rotational scanner, producing a sharp and consistent horizontal slice with each full rotation. This makes it less prone to noise and likely to provide more accurate DBH estimates (LMS511-Product data sheet). The combination of these features—rotating base, stable performance at high tree densities, and sharp 2D resolution—makes the BEE system a strong competitor to LCA-TLS. However, its price, although categorized as low-cost, is still three times higher than LCA-TLS, at under €9000. Given that the average DBH RMSE differed by only 0.233 cm, the price difference should be carefully considered in relation to whether such a small gain in accuracy is worth paying for in practical use, especially when TLS-derived DBH estimation errors typically range between 0.7 cm and 2.7 cm [10,21,22,23,50] and all devices tested in our study fall within this range.

*Tree height*

For tree height, significant deviations were also observed. The Riegl system slightly overestimated height, with a mean bias of approximately 0.122 m. Stonex showed an overestimation of around 0.323 m. In contrast, the LCA-TLS prototype exhibited a strong tendency toward underestimation, with an average bias of −0.912 m. These results reflect a consistent direction of error across the devices, which has important implications for their use in the context of quantitatively accurate data collection.

The LCA-TLS performed slightly worse in this metric, with TH RMSE of 0.992 m and a tendency toward underestimation (bias −0.912). These deviations are statistically significant and are likely due to limitations in canopy top coverage and reduced point density in upper tree sections. This issue appears to be primarily related to the behaviour of the LCA-TLS’s processing algorithm, rather than the ranging capacity of the sensor itself, as the Livox is capable of long-range detection. The algorithm tends to filter out the tree top when an insufficient number of points is detected in the upper crown. However, the scanning pattern may also have contributed to this effect, as the device was mounted in a horizontal position and not tilted upward, which may have further limited point capture in the upper canopy.

The Stonex-based TH was closer to the reference values. The iPhone’s performance in TH estimation was lacking, likely due to the limited range of its sensor and the absence of points in the upper parts of stems and crowns (Figure 19).

The TH results from low-cost systems confirm that tree height estimation remains a challenge for low-cost technologies. In terms of TH estimation, LCA-TLS and LC-MLS lagged behind the accuracy of professional solutions. While LC-MLS reported an RMSE of 2.47 m and a bias of −2.16 m (rRMSE = 12.97%) [49], LCA-TLS showed a lower RMSE of 0.992 and a bias of −0.912. This discrepancy is likely related to differences in point density and data acquisition approach. Although the LCA-TLS employs a more advanced LiDAR sensor-Livox Avia-compared to the LC-MLS’s Livox MID-360, the underlying data collection methods differ significantly.

Although the literature does not define a standardized “acceptable” range of RMSE, results from several studies investigating tree height estimation using various LiDAR-based approaches suggest that RMSE values for tree height derived from TLS typically range from 0.5 m to 1.8 m under favourable conditions [46,48,51,52,53,54,55]. The RMSE values obtained in our study fall well within this empirically established range, further supporting the reliability and consistency of our low-cost TLS-based TH measurements under the given field conditions.

### 5.2. From Accuracy to Application: Evaluating Efficiency, Cost, and User-Friendliness

**Figure 22:**
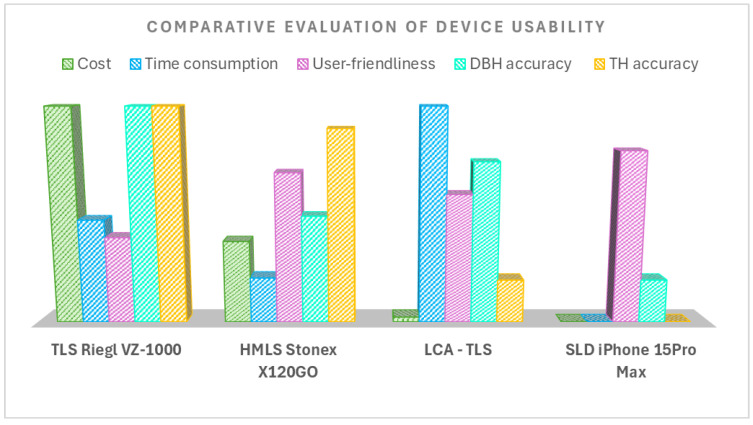
Comparative evaluation of device usability.

With key performance metrics assessed across various terrestrial lidar systems, including our own, the next section turns to practical considerations such as time efficiency, cost, and usability in operational conditions (Figure 22). While the previous section focused on measurement accuracy, it is equally important to assess how these systems perform under real-world operational conditions. HMLS systems are consistently highlighted for their high efficiency, while TLS remains strong in accuracy but is less time-efficient and more costly. It is clear that the use of mobile HMLS systems is advancing rapidly due to their high efficiency, low processing time, and favourable cost. Overall, the advantages of this technology have been clearly demonstrated in numerous studies [10], [21,23,29,34,35,37]. The TLS approach requires more time to acquire the field data, more efforts in processing the data, e.g., the registration of multiple scans and prices remain the highest among terrestrial systems [22]. If we compare professional and low-cost TLSs, we can see that the time needed to create a point cloud with a prototype is slightly longer when following the same multi-position scanning methodology, but on the other hand the input costs are incomparably lower.

In the context, it should be noted that, in addition to differing scanning approaches, the prices of mobile scanning devices in 2024 varied widely depending on the type and features of the device. For a more comprehensive view, other professional SLAM systems, such as the GeoSLAM ZEB Horizon (GeoSLAM Ltd., Nottingham, UK), were available starting at approximately €44,000 (GeoSLAM ZEB Horizon), while high-end TLS devices, such as the Leica RTC360, could cost as much as €84,000 (Leica Geosystems AG, Heerbrugg, Switzerland). On the other hand, solutions using LiDAR sensors in Apple devices were more affordably available, starting at around €900 (Apple Inc., Cupertino, CA, USA). The complete system LCA-TLS was built at a total cost of €2050. In terms of efficiency and purchase price, the iPhone Lidar is highly rated, but the quality of the outputs lags behind other solutions: the RMSE in DBH estimation is high, the height of trees cannot be inferred due to the limited range of the sensor, and the resulting point cloud exhibits a bias due to the absence of SLAM (both objects and their positions within the space). However, it should be noted that this is primarily a software-related limitation, as SLAM-capable applications are available for the iPhone platform (for example RTAB-Map (IntRoLab, Université de Sherbrooke, Sherbrooke, QC, Canada)), but they were not used in this study, as the objective was to assess the device’s native performance without external software enhancements.

### 5.3. From Challenges to Opportunities: Technical Limits, Future Research, and Real-World Potential

Although a TLS provides detailed 3D data, its static nature, occlusion effects, long scanning times, and high cost limit its effectiveness for data collection in larger and heterogeneous forest stands, motivating the use of mobile alternatives such as handheld MLS systems [49]. On the other hand, while MLS can achieve good results in certain parameters, the accuracy and quality of the resulting point cloud, particularly in complex or long-range scanning scenarios, is generally not comparable to that of TLSs [10,21].

It should be noted that in our work, we aim to strike a balance between cost and performance. While the use of a TLS would be appropriate in many cases, its high acquisition cost is unaffordable for many users. For this reason, we seek to offer an alternative that addresses the limitations of mobile technologies while retaining the benefits of TLS at a significantly lower acquisition cost.

From the perspective of the overall functionality of the LCA-TLS prototype and the results achieved, we can conclude that its operation and overall usability are, in terms of time and user experience, almost comparable to professional TLS systems. As demonstrated, the accuracy of the derived parameters is promising (Table 5 and Table 6—DBH a TH summary statistic).

Although the presented results are based on plantation conditions, similar issues may arise in natural forest stands, along with specific challenges related to higher structural complexity. It should be noted that such limitations can also affect standard TLS systems. In real forest environments, where noise levels are higher and vegetation is denser, there is an increased risk that the processing algorithm may omit thinner trees or objects with weaker geometric definition. We plan to investigate this issue further, as it represents one of the key areas we aim to address in future work.

Compared to other low-cost measurement methods such as close-range photogrammetry, the LCA-TLS scanner is an active device that enables direct and straightforward measurement without relying on ambient light conditions. Unlike photogrammetry, which is highly sensitive to lighting and often struggles with shadows and contrast in forest environments, the scanner provides stable results even under reduced visibility in the understory or in poor lighting conditions [48,56].

A deeper look into the technical configuration reveals further areas of both strength and limitation in the LCA-TLS. Compared with high cost of commercial laser scanners, the LCA-TLS has its own limitations. One of the main ones is its field of view (FOV). The integrated Livox Avia sensor has an FOV of 70.4° horizontally and 77.2° vertically, whereas the RIEGL VZ-1000 offers an FOV of 360° horizontally and 100° vertically, and the Stonex X120GO provides 360° horizontally and approximately 90–100° vertically (value not precisely specified) (Livox Avia LiDAR sensor). An interesting advantage is the Livox Avia sensor’s ability to generate higher point density in the centre of the FOV (within a radius of ±10°), which represents a significant technological benefit. Despite having a smaller FOV compared to the other assessed devices, it can achieve high point density, especially in the central parts of the scene (Livox Avia User Manual).

A second limitation is the fact that, at this stage of development, the prototype is not capable of automated rotation, which marks a notable difference compared to standard TLS systems, where rotation is an integral part of the data acquisition process. Since the device does not yet support automated rotation, its current limitations also create opportunities for further development, particularly toward the implementation of a rotating mechanism. At this stage of prototype development, colourized point cloud visualization is not implemented, as it would require an additional sensor, such as a camera. However, for many tasks, such data enhancement may not be essential. Additionally, the software environment has not yet been designed with user accessibility in mind, which presents another area for future improvement.

Despite these limitations, we consider the price-to-performance ratio to be very user-friendly, particularly for tasks such as forest stand inventory, where the device could significantly reduce labour requirements through automated tree counting or DBH measurements, thus accelerating the entire process. Conventional forest inventory methods can take between 5 and 9.5 h per hectare [57,58], depending on whether only DBH or other dendrometric parameters are measured, which corresponds to approximately 27 to 57 min for an area of 30 × 30 m (which is approximately the size of one of our research plots). In contrast, terrestrial LiDAR scanning provides substantial added value by capturing a comprehensive dataset rapidly, enabling multiple tree parameters to be extracted from a point cloud. In this context, the prototype represents a potentially valuable investment for smaller companies, individual users, or as an affordable first-step tool that fits well with what is already used and helps improve data collection efficiency in forestry.

## 6. Conclusions

This study evaluated the performance of a low-cost TLS prototype, LCA-TLS. Designed as a fully open-source and open-hardware solution, all components are easily obtainable, and the complete assembly and workflow documentation is publicly available via Zenodo, enabling effortless reproduction and adaptation. The testing included two experiments: one conducted under controlled indoor conditions, and another carried out in a Paulownia plantation. In both cases, the prototype was compared with commercially used devices that differed in precision and price. The results show that, despite its simple design and low cost (€2050), the LCA-TLS achieved competitive accuracy in measuring diameter at breast height (RMSE 1.5 cm) and acceptable accuracy in tree height estimation (RMSE less than 1 m). By bridging the gap between cost and accuracy, it addresses a key community need for affordable precision tools with a compact and lightweight design. The system is well suited for monitoring small forest plots, where the time required for data acquisition is manageable. Although the data processing interface is still under development, the prototype already demonstrates potential as an affordable tool for individual users, small companies, or research groups. Further development will focus on improving canopy top detection and adding an automated rotation mechanism to increase efficiency and usability in more complex environments. Overall, the LCA-TLS offers a practical balance between measurement accuracy and operational costs in modern forest data collection. These findings confirm that low-cost, open-source TLS solutions can play a relevant role in forest monitoring and serve as a valuable entry point for broader adoption of 3D technologies in forestry practice.

## Figures and Tables

**Table 1:** LIVOX AVIA basic specification (source https://www.livoxtech.com/avia/specs (accesses on 8 October 2025)).

Laser Wavelength	905 nm
Number of returns	3
Detection Range	190 m @ 10% reflectivity
(@ 100 klx)	230 m @ 20% reflectivity
	320 m @ 80% reflectivity
Detection Range	190 m @ 10% reflectivity
(@ 0 klx)	260 m @ 20% reflectivity
	450 m @ 80% reflectivity
FOV	Non-repetitve scanning pattern
	70.4° (Horizontal) × 77.2° (Vertical)
	Repetitve line scanning
	70.4° (Horizontal) × 4.5° (Vertical)
Range Precision	2 cm
(1σ @ 20 m)	
Angular Precision	<0.05º
(1σ)	

**Table 2:** Estimated tube diameters and corresponding errors (Diff., in cm) for each device, compared to reference measurements. The table includes raw estimated values, error values (Diff. = Estimated − Reference), and summary statistics such as RMSE and Bias for each device.

ID	Reference Diameter	iPhone			LCA-TLS			Riegl			Stonex		
		Detec.	Est.	Diff.	Detec	Est.	Diff.	Detec	Est.	Diff.	Detec	Est.	Diff.
ID01	11.10	yes	10.30	−0.80	yes	11.40	0.30	yes	11.50	0.40	yes	11.90	0.80
ID02	16.00	yes	16.70	0.70	yes	16.80	0.80	yes	16.50	0.50	yes	16.80	0.80
ID03	16.00	yes	16.90	0.90	yes	16.40	0.40	yes	16.80	0.80	yes	16.70	0.70
ID04	16.00	yes	16.30	0.30	yes	16.80	0.80	yes	16.40	0.40	yes	16.90	0.90
ID05	16.00	yes	16.20	0.20	yes	16.30	0.30	yes	16.70	0.70	yes	17.00	1.00
ID06	16.00	yes	16.70	0.70	yes	16.50	0.50	yes	16.80	0.80	yes	16.90	0.90
ID07	16.00	yes	16.80	0.80	yes	16.60	0.60	yes	16.75	0.75	yes	16.80	0.80
ID08	25.30	yes	27.40	2.10	yes	26.40	1.10	yes	25.80	0.50	yes	26.20	0.90
ID09	25.30	yes	28.30	3.00	yes	26.60	1.30	yes	26.00	0.70	yes	26.40	1.10
ID10	50.50	yes	54.31	3.81	yes	52.70	2.20	yes	51.10	0.60	yes	53.10	2.60
ID11	2.60	no	0	−2.60 *	yes	4.80	2.20	yes	3.15	0.55	yes	5.20	2.60
ID12	2.60	no	0	−2.60 *	yes	4.40	1.80	yes	3.08	0.48	yes	4.90	2.30
RMSE (cm)		1.76			1.23			0.62			1.47		
Bias (cm)		1.17			1.03			0.60			1.28		

* Undetected objects were excluded from the RMSE and Bias calculations.

**Table 3:** Estimated tube heights and associated errors (Diff., in cm) for each device compared to reference measurements. The table presents raw estimated values, differences from the reference (Diff. = Estimated − Reference), and summary statistics including RMSE and Bias for each device.

ID	Ref. Height	iPhone			LCA-TLS			Riegl			Stonex		
		Detec.	Est.	Diff.	Detec.	Est.	Diff.	Detec	Est.	Diff.	Detec.	Est.	Diff.
ID01	145.00	yes	152.00	7.00	yes	149.00	4.00	yes	148.00	3.00	yes	151.00	6.00
ID02	100.00	yes	102.00	2.00	yes	105.00	5.00	yes	101.00	1.00	yes	108.00	8.00
ID03	100.00	yes	108.00	8.00	yes	104.00	4.00	yes	100.80	0.80	yes	102.00	2.00
ID04	100.00	yes	105.00	5.00	yes	100.80	0.80	yes	102.00	2.00	yes	102.00	2.00
ID05	100.00	yes	101.00	1.00	yes	102.00	2.00	yes	104.00	4.00	yes	104.00	4.00
ID06	100.00	yes	102.40	2.40	yes	103.00	3.00	yes	102.00	2.00	yes	105.40	5.40
ID07	100.00	yes	105.00	5.00	yes	104.00	4.00	yes	102.50	2.50	yes	103.00	3.00
ID08	180.00	no	0	−180 *	yes	183.00	3.00	yes	182.00	2.00	yes	185.00	5.00
ID09	158.00	no	0	−158 *	yes	161.00	3.00	yes	159.00	1.00	yes	164.00	6.00
ID10	216.00	yes	186.00	−30.00	yes	224.00	8.00	yes	218.00	2.00	yes	227.00	11.00
ID11	290.00	yes	224.00	−66.00	yes	301.00	11.00	yes	292.00	2.00	yes	305.00	15.00
ID12	290.00	yes	195.00	−95.00	yes	296.00	6.00	yes	294.00	4.00	yes	299.00	9.00
RMSE (cm)		38.02			5.21			2.41			7.36		
Bias (cm)		−16.06			4.48			2.19			6.37		

* Undetected objects were excluded from the RMSE and Bias calculations.

**Table 4:** Descriptive statistics of DBH estimation for Forest Condition Experiment.

DBH		Device			
		TLS Riegl	HMLS Stonex	LCA-TLS	SLD iPhone
R^2^		0.973	0.944	0.943	0.920
RMSE	(cm)	0.754	1.317	1.503	2.007
rRMSE	(%)	2.943	5.144	5.872	7.840
Bias	(cm)	0.470	1.012	1.241	1.739
rBias	(%)	1.835	3.952	4.847	6.790
Reference_max_	(cm)	39.500			
Reference_min_	(cm)	17.300			
Estimated_max_	(cm)	40.201	40.790	41.700	43.157
Estimated_min_	(cm)	17.988	18.860	17.950	19.553
Error_max_	(cm)	1.662	3.146	3.442	4.110
Error_min_	(cm)	−1.353	−1.366	−0.985	−1.008
N	(tree)	151	151	151	151
TDR	(%)	100	100	100	100

**Table 5:** Descriptive statistics of TH estimation for Forest Condition Experiment.

TH		Device		
		Riegl	Stonex	LCA-TLS
R^2^		0.956	0.927	0.902
RMSE	(m)	0.290	0.468	0.992
rRMSE	(%)	1.847	2.977	6.316
Bias	(m)	0.122	0.323	−0.912
rBias	(%)	0.776	2.058	−5.805
Reference_max_	(m)	17.694		
Reference_min_	(m)	11.425		
Estimated_max_	(m)	17.805	17.960	16.250
Estimated_min_	(m)	11.860	11.570	10.750
Error_max_	(m)	0.650	1.026	−0.112
Error_min_	(m)	−0.558	−0.574	−1.819
N	(tree)	151	151	151

**Table 6:** The overall comparison of the tested devices in terms of time requirements, cost, ease of use, and accuracy.

Device	Data Collection (Approx.)	Post-Processing (Approx.)	Price (Approx.)	User Friendliness (1–5 Points) *	Accuracy (RMSE)	
					DBH (cm)	TH (m)
TLS Riegl VZ-1000	21 min	26 min	€90,000 to €130,000	2.1	0.754	0.290
HMLS Stonex X120GO	5 min	20 min	€30,000 to €35,000	2.6	1.317	0.468
LCA-TLS	40 min	49 min	€2050	2.4	1.503	0.992
SLD iPhone 15 Pro Max	8 min	no	€850 to €1200	3.2	2.007	-

* A higher value corresponds to a more favourable user evaluation.

**Figure A1:**
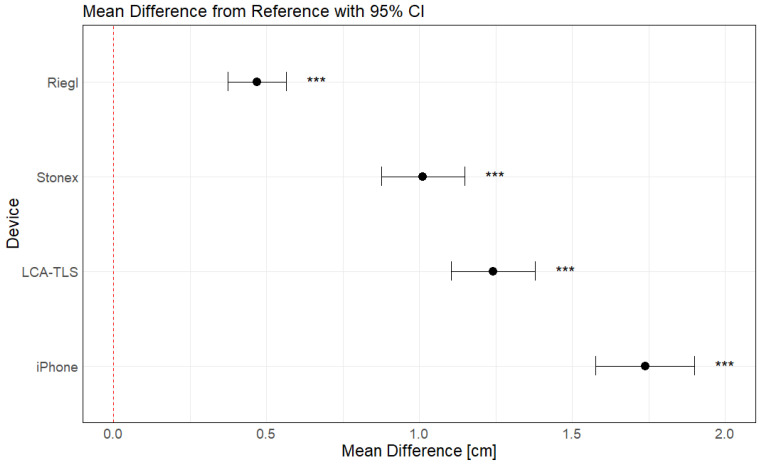
Paired *t*-test Results (DBH): Mean Differences from Reference with 95% CI. Significance code: *** *p* < 0.001.

**Figure A2:**
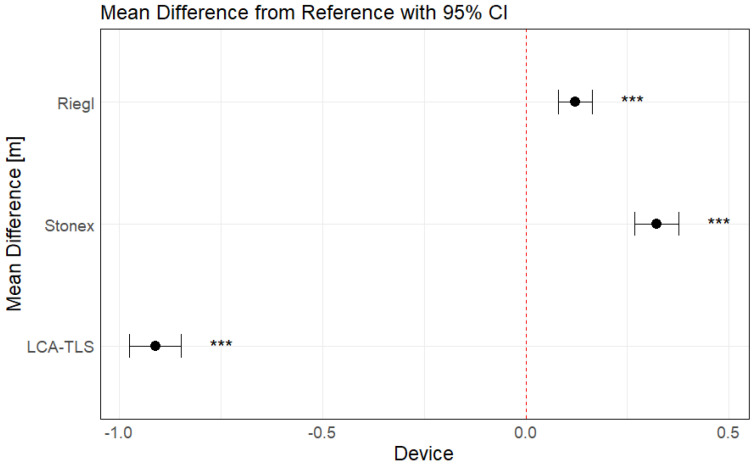
Paired *t*-test Results (TH): Mean Differences from Reference with 95% CI. Significance code: *** *p* < 0.001.

**Figure A3:**
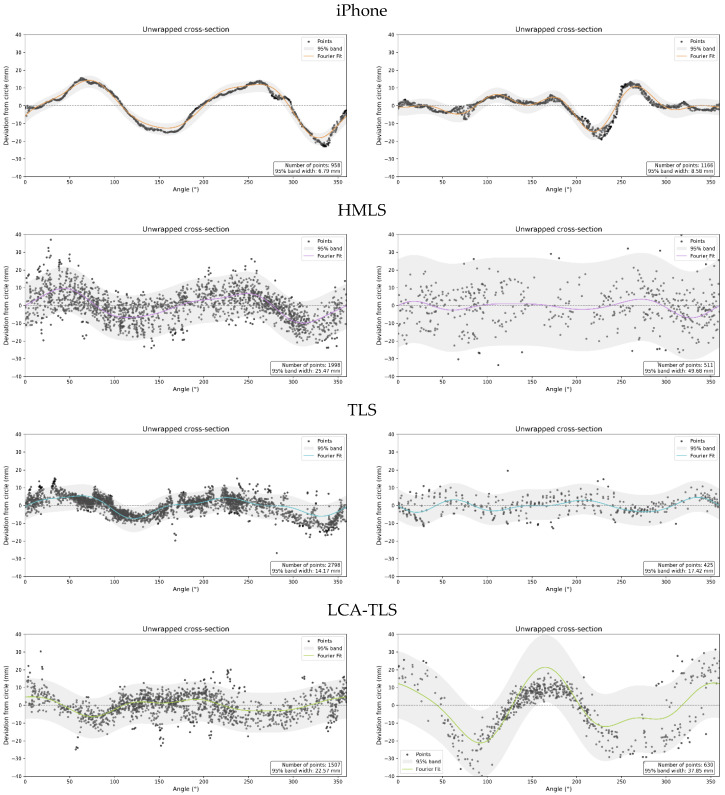
Unwrapped cross-sections at 1.3 m above ground for the four tested devices, showing radial deviations from the fitted reference circle, number of points, and 95% residual band width: tube (**Left**) and tree (**Right**). Different colors represent individual devices: blue—TLS, purple—HMLS, green—LCA-TLS, and orange—SLD.

## Data Availability

The data presented in this study are available on request from the corresponding author.

## Appendix A

### Appendix A.1

**Table A1:** Analysis of variance results (DBH).

Term	Df	Sum of Squares	Mean Square	F-Value	*p*-Value	Significance
Device	3	125.6	41.86	59.74	<2 × 10^−16^	***
Residuals	600	232.4	0.38			

Significance codes: *** *p* < 0.001.

### Appendix A.2

**Table A2:** Tukey HSD test results for pairwise comparison of devices (DBH estimation).

Comparison *	Mean Diff. (cm)	Lower CI (cm)	Upper CI (cm)	Adjusted *p*-Value	Significance
HMLS–TLS	0.54201	0.29382	0.79019	1.7 × 10^−7^	***
LCA-TLS–TLS	0.77126	0.52307	1.01944	4.48 × 10^−10^	***
SLD–TLS	1.26879	1.02061	1.51697	4.48 × 10^−10^	***
LCA-TLS–HMLS	0.22925	−0.01893	0.47743	8.21 × 10^−2^	
SLD–HMLS	0.72678	0.4786	0.97497	4.49 × 10^−10^	***
SLD–LCA-TLS	0.49753	0.24935	0.74572	1.96 × 10^−6^	***

Significance code: *** *p* < 0.001. * SLD-iPhone 15 Pro Max. HMLS—Stonex X120GO. TLS—Riegl VZ-1000.

### Appendix A.4

**Table A3:** Results of paired *t*-tests for diameter at breast height (DBH) compared to manual reference (95% confidence intervals).

Device	Mean Difference (cm)	Lower Bound (95% CI)	Upper Bound (95% CI)	*p*-Value	Significance
Riegl	0.46988	0.37484	0.56493	9.49 × 10^−18^	***
LCA-TLS	1.24114	1.10426	1.37801	4.21 × 10^−39^	***
Stonex	1.01189	0.87586	1.14792	7.37 × 10^−31^	***
iPhone	1.73867	1.57683	1.90052	4.88 × 10^−47^	***

Significance code: *** *p* < 0.001.

### Appendix A.5

**Table A4:** Analysis of variance results (TH).

Term	Df	Sum of Squares	Mean Square	F-Value	*p*-Value	Significance
Device	2	133.42	66.71	591.9	2 × 10^−16^	***
Residuals	453	51.05	0.11			

Significance code: *** *p* < 0.001.

### Appendix A.6

**Table A5:** Tukey HSD test results for pairwise comparison of devices (TH estimation).

Comparison *	Mean Diff. (cm)	Lower CI (cm)	Upper CI (cm)	Adjusted *p*-Value	Significance
HMLS–TLS	0.20142	0.11087	0.29197	8 × 10^−7^	***
LCA-TLS–TLS	–1.03340	–1.12395	–0.94285	<0.001	***
LCA-TLS–HMLS	–1.23482	–1.32537	–1.14427	<0.001	***

Significance code: *** *p* < 0.001. * SLD-iPhone 15Pro Max. HMLS–Stonex X120GO. TLS–Riegl VZ-1000.

### Appendix A.8

**Table A6:** Results of paired *t*-tests for tree height (TH) compared to manual reference (95% confidence intervals).

Device	Mean Difference (m)	Lower Bound (95% CI)	Upper Bound (95% CI)	*p*-Value	Significance
Riegl	0.12179	0.07947	0.1641	6.50 × 10^−8^	***
Stonex	0.32321	0.2689	0.37752	4.53 × 10^−23^	***
LCA-TLS	–0.91161	–0.97440	–0.84882	5.41 × 10^−63^	***

Significance code: *** *p* < 0.001.
